# Design and Development of Novel Urea, Sulfonyltriurea, and Sulfonamide Derivatives as Potential Inhibitors of Sphingosine Kinase 1

**DOI:** 10.3390/ph13060118

**Published:** 2020-06-09

**Authors:** Sonam Roy, Amarjyoti Das Mahapatra, Taj Mohammad, Preeti Gupta, Mohamed F. Alajmi, Afzal Hussain, Md. Tabish Rehman, Bhaskar Datta, Md. Imtaiyaz Hassan

**Affiliations:** 1 Centre for Interdisciplinary Research in Basic Sciences, Jamia Millia Islamia, Jamia Nagar, New Delhi 110025, India; roysonam12@gmail.com (S.R.); taj144796@st.jmi.ac.in (T.M.); fun.preets@gmail.com (P.G.); 2 Department of Chemistry, Indian Institute of Technology, Palaj, Gandhinagar, Gujarat 382355, India; amarjyoti.mahapatra@iitgn.ac.in; 3 Department of Pharmacognosy, College of Pharmacy, King Saud University, Riyadh 11451, Saudi Arabia; malajmii@ksu.edu.sa (M.F.A.); afzal.hussain.amu@gmail.com (A.H.); m.tabish.rehman@gmail.com (M.T.R.)

**Keywords:** sphingosine kinase-1, sphingosine-1-phosphate, cancer therapy, enzyme inhibition, kinase inhibitors, molecular docking, drug design and discovery

## Abstract

Sphingosine kinase 1 (SphK1) is one of the well-studied drug targets for cancer and inflammatory diseases. Recently discovered small-molecule inhibitors of SphK1 have been recommended in cancer therapeutics; however, selectivity and potency of first-generation inhibitors are great challenge. In search of effective SphK1 inhibitors, a set of small molecules have been designed and synthesized bearing urea, sulfonylurea, sulfonamide, and sulfonyltriurea groups. The binding affinity of these inhibitors was measured by fluorescence-binding assay and isothermal titration calorimetry. Compounds **1**, **5**, **6**, and **7** showed an admirable binding affinity to the SphK1 in the sub-micromolar range and significantly inhibited SphK1 activity with admirable IC_50_ values. Molecular docking studies revealed that these compounds fit well into the sphingosine binding pocket of SphK1 and formed significant number of hydrogen bonds and van der Waals interactions. These molecules may be exploited as potent and selective inhibitors of SphK1 that could be implicated in cancer therapeutics after the required *in vivo* validation.

## 1. Introduction

Lipid kinases represent a prominent class of regulatory enzymes [1]. Dysregulation of lipid kinases is frequently associated with several diseases including cancers, diabetes, and cardiovascular disorders [2,3,4,5]. Similarly, cognate lipid substrates for the kinases, such as phosphatidylinositol-3,4,5-triphosphate, phosphatidic acid, and sphingolipids, are distinctive signaling molecules impacting verities of cellular processes [6,7,8]. Among lipid kinases, phosphoinositide 3-kinase (PI3K) plays an important role in mitogenic signaling and cell survival, cytoskeletal remodeling, metabolic control, and vesicular trafficking [9]. Both PI3K and phosphatidylinositol 4-phosphate 5-kinase (PIP5K) are potential mediators of Rho/Rac functions [10]. Diacylglycerol kinases (DGKs) stimulate DNA synthesis and subsequently modulate the activity of several enzymes including phosphatidylinositol 5-kinases (PI-5-K), protein kinase C zeta (PKCζ), and Ras-GAP [11,12,13].

The sphingosine kinases (SphKs) are a distinct group of lipid kinases bearing no similarity to PI3Ks, but possess a protein fold similar to DGKs, NAD kinases, ceramide kinases, and PFKs. These play an important role in maintaining homeostasis in the cellular environment by controlling the levels of three core sphingolipids forming the sphingolipid rheostat [14]. Up or down-regulation of DGKs has been linked with several pathological conditions including cancer, epilepsy, Parkinson’s disease, bipolar disorders, cardiac hypertrophy, type II diabetes, and hypertension [15]. Sphingosine-1-phosphate (S1P) is a prominent representative of the membrane-derived class of sphingolipids and is intracellularly produced by ATP-dependent phosphorylation of sphingosine by SphKs [16,17]. S1P functions both as a lipid secondary messenger and a ligand for G-protein coupled endothelial-derived receptor 1 (Edg-1) [18,19]. S1P and its pre-cursor ceramide provide the support that balances tumor growth versus tumor suppression [20]. Sphingosine, S1P, and ceramide have been implicated in multiple signaling pathways especially pertinent to stress response [21,22,23]. S1P has been connected to several diseases including different types of cancer, diabetes, chronic inflammatory conditions such as atherosclerosis, and asthma [24,25,26,27].

The two isoforms of SphKs, namely SphK1 and SphK2, exert distinct functions inside the cell. Nevertheless, as suggested by knockout studies, they appear to compensate for each other during embryonic development. SphK1 is involved in cell survival pathways, while SphK2 is pro-apoptotic and induces apoptosis in cells [28].

Overexpression of SphK1 is correlated with tumor growth and metastasis [29,30,31]. SphK1 inhibition is considered as an attractive strategy for cancer therapeutics [32,33,34,35]. The crystal structure of SphK1 has been used in structure-based drug design strategies to discover small-molecule inhibitors for therapeutic uses. Co-crystallized SphK1-selective inhibitors, such as PF-543 and SKI-II, have provided insights into effective modes of binding, thereby helping in the discovery of next-generation inhibitors [36,37]. Notably, SK1-I selectively inhibits SphK1 and has been shown to effectively reduce the growth and survival of human leukemia U937 cells in vitro [38]. An immunosuppressant Fingolimod, upon phosphorylation by SphK2, acts as a functional antagonist of S1P receptors, S1P, and is an approved drug for the treatment of multiple sclerosis [39]. Discovery of more potent and selective SphK1 inhibitors could lead to the development of new therapy for the treatment of cancer and/or immune-mediated diseases.

**Figure 1:**
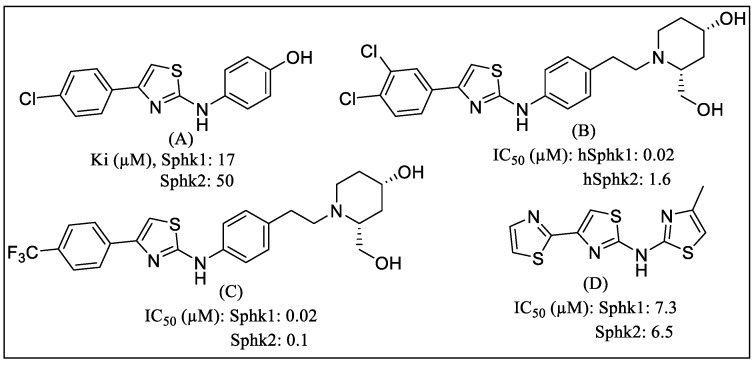
Chemical structure of thiazole-based sphingosine kinase 1 inhibitor.

**Figure 2:**
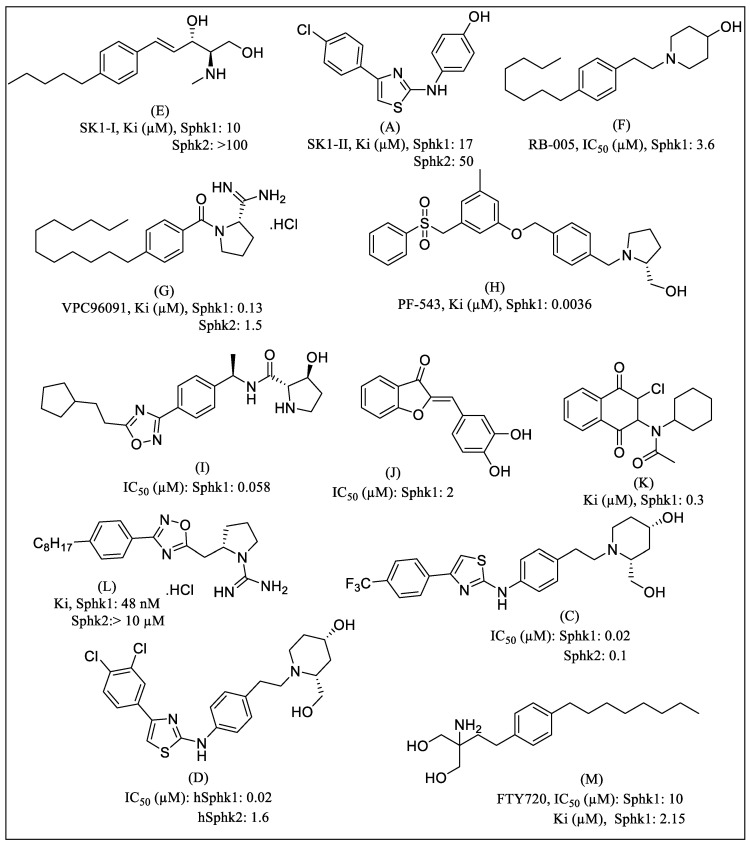
Major structural scaffolds reported as inhibitors of SphK1.

A diverse range of scaffolds has been incorporated in selective SphK1 inhibitors, including derivatives of guanidine [40], amidine [41], quinolin-2-one-pyrimidine hybrids [42], 2-epi-jaspineB [43], 1,2,3-triazole [44], 2-amino benzothiazole [45], pyrazole-5-carbohydrazide [46], sulfonylurea [47], and carbamate [48]. Our group is exploring the scope of urea, sulfonylurea, and bioisosteres in novel chemical biology contexts [49,50]. Substituted thiazoles have been reported to possess sphingosine kinase 1 inhibitory properties (Figure 1) [45,51]. Similarly, amidine, carbamate, and guanidine-based subtype-selective sphingosine kinase 1 inhibitor have been developed (Figure 2) [52,53,54,55,56,57,58]. These molecules have inspired us to design urea, sulfonylurea, sulfonyltriurea, sulfonamide, and N-monosubstituted urea-based small molecules for screening against the active site pocket of SphK1. Accordingly, we have tested a small set of molecules, encompassing the groups of our interest, towards inhibition of sphingosine kinase 1.

In the current study, a set of 11 novel small molecules possessing urea, sulfonylurea, sulfonamide, and sulfonyltriurea groups were synthesized, characterized, and screened for their inhibitory activity against SphK1. Fluorescence binding studies, isothermal titration calorimetry (ITC), and molecular docking were performed to gain insights into the binding mechanism.

## 2. Results

### 2.1. Chemistry

**Figure 3:**
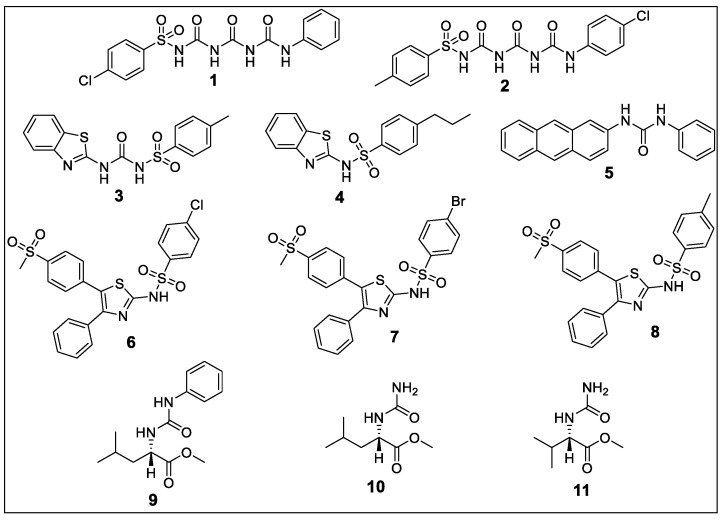
Structures of all synthesized compounds (**1** to **11**).

Diverse groups of small molecules possessing different scaffolds including diarylsulfonyltriurea (Compounds **1** and **2**), benzothiazole-based sulfonylurea and sulfonamide (Compounds **3** and **4**), anthracene-based urea (Compound **5**), diaryl-substituted thiazole-based sulfonamide (Compounds **6**, **7**, and **8**), and amino acid-based urea and N-monosubstituted urea derivatives (Compounds **9**, **10**, and **11**) were synthesized (Figure 3) according to the reported methods. All molecules were characterized by different spectroscopic techniques such as ^1^H NMR, ^13^C NMR, and LC-MS mass spectrometry (Figure S1). The mass spectra of all the compounds agreed with the calculated values, thus confirming the formation of desired compounds. All compounds were confirmed as having ≥95% purity before performing any biological assay.

Characterization of Synthesized Compounds (C**1** to C**11**; Figure S1)

#### 2.1.1. 4-chloro-*N*-(((phenylcarbamoyl)carbamoyl)carbamoyl)benzenesulfonamide (**1**)

Yield 27%, ^1^H NMR (DMSO-d6, 500 MHz) δ: 10.06 (s, 1H, NH), 9.91 (s, 1H, NH), 9.70 (s, 1H, NH), 7.99 (d, 2H, *J* = 8.4 Hz), 7.73 (d, 2H, *J* = 8.4 Hz), 7.47 (d, 2H, *J* = 7.8 Hz), 7.34 (t, 2H, *J* = 7.8 Hz), 7.10 (t, 1H, *J* = 7.3 Hz), ^13^C NMR (DMSO-d6, 125 MHz) δ: 152.1, 151.0, 150.3, 138.9, 137.9, 130.1, 129.7, 129.5, 124.3, 120.1, MS (ESI, *m/z*) calculated for C_15_H_13_ClN_4_O_5_S, [M+H]^+^: 397.0368, found: 397.0368. 

#### 2.1.2. *N*-((((4-chlorophenyl)carbamoyl)carbamoyl)carbamoyl)-4-methylbenzenesulfonamide (**2**)

Yield 25%, ^1^H NMR (DMSO-d_6_, 500 MHz) δ: 9.98 (s, 1H, NH), 9.83 (s, 1H, NH), 9.74 (s, 1H, NH), 7.89–7.87 (m, 2H), 7.51 (d, 2H, *J* = 9 Hz), 7.46 (d, 2H, *J*= 8 Hz), 7.39 (d, 2H, *J* = 8.5 Hz), 2.42 (s, 3H, CH_3_); ^13^C NMR (DMSO-d_6_, 125 MHz) δ: 151.9, 150.9, 149.6, 144.9, 136.9, 136.7, 130.1, 129.3, 128.2, 128.0, 121.8, 21.6. MS (ESI, *m/z*) calculated for C_16_H_15_ClN_4_O_5_S, [M+H]^+^: 411.0524, found: 411.0750.

#### 2.1.3. *N*-(benzo[d]thiazol-2-ylcarbamoyl)-4-methylbenzenesulfonamide (**3**)

Yield 30%, ^1^H NMR (DMSO-d_6_, 500 MHz) δ: 7.91–7.85 (m, 3H), 7.57 (d, 1H, *J* = 6.5 Hz), 7.46–7.40 (m, 3H), 7.27 (t, 1H, *J* = 7.5 Hz), 5.76 (s, 1H, NH), 2.41 (s, 3H, CH_3_); ^13^C NMR (DMSO-d6, 125 MHz) δ: 144.4, 137.4, 130.0, 128.1, 127.0, 123.9, 122.5, 21.5; MS (ESI, *m/z*) calculated for C_15_H_13_N_3_O_3_S_2_ [M+H]^+^:348.0471, found: 348.0476.

#### 2.1.4. *N*-(benzo[d]thiazol-2-yl)-4-propylbenzenesulfonamide (**4**)

Yield 40%, ^1^H NMR (DMSO-d6, 500 MHz) δ: 7.79 (t, 3H, *J* = 8 Hz), 7.38–7.37 (m, 3H), 7.31 (d, 1H, *J* = 7.5 Hz), 7.25 (t, 1H, *J* = 7.5 Hz), 2.61–2.58 (m, 2H), 1.60–1.56 (m, 2H), 0.88–0.85 (m, 3H); ^13^C NMR (DMSO-d6, 125 MHz) δ: 167.3, 147.6, 139.9, 129.4, 127.7, 126.3, 125.3, 124.1, 123.2, 113.3, 37.4, 24.2, 14.0; MS (ESI, *m/z*) calculated for C_16_H_16_N_2_O_2_S_2_, [M+H]+: 333.0726, found: 333.0738.

#### 2.1.5. 1-(anthracen-2-yl)-3-phenylurea (**5**)

Yield 20%, ^1^H NMR (DMSO-d_6_, 500 MHz) δ: 8.95 (s, 1H), 8.79 (s, 1H), 8.48 (s, 1H), 8.41 (s, 1H), 8.28 (s, 1H), 8.04 (t, 3H, *J* = 8.5), 7.52–7.43 (m, 5H), 7.33 (t, 2H, *J* = 7.5), 7.02 (t, 1H, *J* = 7.5); ^13^C NMR (DMSO-d6, 125 MHz) δ:153.2, 140.0, 137.1, 132.4, 132.2, 130.6, 129.4, 129.3, 128.6, 128.5, 128.1, 126.3, 126.1, 125.2, 124.8, 122.6, 121.5, 118.9, 112.4; MS (ESI, *m/z*) calculated for C_21_H_16_N_2_O, [M+H]^+^: 313.1335 found: 313.1360.

#### 2.1.6. 4-chloro-*N*-(5-(4-(methylsulfonyl)phenyl)-4-phenylthiazol-2-yl)benzenesulfonamide (**6**)

Yield 55%, ^1^H NMR (CDCl_3_, 500 MHz): δ 7.92 (d, 2H, *J* = 7.5 Hz), 7.84 (d, 2H, *J* = 8.0 Hz), 7.53 (d, 2H, *J* = 8.0 Hz), 7.46 (d, 2H, *J* = 8.0 Hz), 7.35 – 7.33 (m, 4H), 7.22 (d, 2H, *J* = 6.5 Hz), 3.06 (s, 3H),^13^C NMR (CDCl_3_, 125 MHz): 167.2, 140.8, 140.6, 138.3, 134.4, 130.1, 129.9, 129.4, 129.2, 129.2, 129.1, 128.9, 127.9, 127.6, 122.4, 44.2,MS (ESI, *m/z*) calculated for C_22_H_17_ClN_2_O_4_S_3_, [M+H]^+^: 505.0112, found: 505.0101 and [M+2]^+^: 507.0057.

#### 2.1.7. 4-bromo-*N*-(5-(4-(methylsulfonyl)phenyl)-4-phenylthiazol-2-yl)benzenesulfonamide (**7**)

Yield 51%, ^1^H NMR (DMSO-d 6, 500 MHz): δ 13.42 (brs, 1H), 7.92 (d, 2H, *J* = 8.5 Hz), 7.84–7.80 (m, 4H), 7.61 (d, 2H, *J* = 8.0 Hz), 7.40–7.39 (m, 3H), 7.29–7.28 (m, 2H), 3.26 (s, 3H), ^13^C NMR (DMSO-d_6_, 125 MHz): 166.80, 141.9, 141.8, 134.2, 132.7, 130.4, 130.2, 129.7, 129.5, 129.4, 128.5, 127.6, 126.5, 121.4, 43.7, MS (ESI, *m/z*) calculated for C_22_H_17_BrN_2_O_4_S_3_, [M+H]^+^: 548.9607, found: 548.9610 and [M+2]^+^: 550.9595.

#### 2.1.8. 4-methyl-*N*-(5-(4-(methylsulfonyl)phenyl)-4-phenylthiazol-2-yl)benzenesulfonamide (**8**)

Yield 60%, ^1^H NMR (DMSO-d_6_, 500 MHz): δ 13.26 (brs, 1H), 7.91 (d, 2H, *J* = 8.0 Hz), 7.79 (d, 2H, *J* = 8.0 Hz), 7.60 (d, 2H, *J* = 8.5 Hz), 7.40–7.39 (m, 5H), 7.27–7.27 (m, 2H), 3.26 (s, 3H), 2.39 (s, 3H),^13^C NMR (DMSO-d_6_, 125 MHz): 143.1, 141.7, 139.7, 130.4, 130.3, 130.0, 129.7, 129.5, 129.3, 127.6, 126.5, 43.7, 21.4, MS (ESI, *m/z*) calculated for C_23_H_20_N_2_O_4_S_3_, [M+H]^+^: 485.0658, found: 485.0688.

#### 2.1.9. Methyl (phenylcarbamoyl)-l-leucinate (**9**)

Yield 38%, ^1^H NMR (DMSO-d_6_, 500 MHz) δ: 8.53 (s, 1H, NH), 7.38 (d, 2H, *J* = 7.5 Hz), 7.24 (t, 2H, *J* = 8 Hz), 6.92 (t, 1H, *J* = 7.5 Hz), 6.52 (d, 1H, *J* = 8 Hz), 4.30–4.25 (m, 1H), 3.67 (s, 3H), 1.74–1.63 (m, 1H), 1.56–1.53 (m, 2H), 0.93 (d, 6H, *J* = 6.5 Hz); ^13^C NMR (DMSO-d6, 125 MHz) δ: 174.3, 155.3, 140.5, 129.2, 121.8, 118.1, 52.3, 51.2, 41.2, 24.8, 23.2, 22.0; MS (ESI, *m/z*) calculated for: C_14_H_20_N_2_O_3_, [M + Na]^+^: 287.1371, found: 287.1363.

#### 2.1.10. Methyl carbamoyl-l-leucinate (**10**)

Yield 48%, ^1^H NMR (DMSO-d6, 500 MHz) δ: 6.31 (d, 1H, *J* = 8 Hz), 5.56 (s, 2H), 4.18–4.13 (m, 1H), 3.62 (s, 3H), 1.69–1.59 (m, 1H), 1.46–1.43 (m, 2H), 0.91–0.87 (m, 6H); ^13^C NMR (DMSO-d6, 125 MHz) δ: 174.7, 158.6, 52.1, 51.3, 41.3, 24.7, 23.2, 22.0; MS (ESI, *m/z*) calculated for C_8_H_16_N_2_O_3_, [M+Na]^+^: 211.1059, found: 211.1049.

#### 2.1.11. Methyl carbamoyl-l-valinate (**11**)

Yield 52%, ^1^H NMR (DMSO-d6, 500 MHz) δ: 6.30 (d, 1H, *J* = 8.5), 5.61 (s, 2H), 4.07–4.05 (m, 1H), 3.64 (s, 3H), 2.03–1.94 (s, 1H), 0.88–0.84 (m, 6H); ^13^C NMR (DMSO-d6, 125 MHz) δ: 173.7, 158.8, 58.1, 52.0, 30.9, 19.5, 18.3; MS (ESI, *m/z*) calculated for C_7_H_14_N_2_O_3_, [M+Na]^+^: 197.0902, found: 197.0901.

### 2.2. Biological Evaluations

#### 2.2.1. Fluorescence Binding Studies

**Figure 4:**
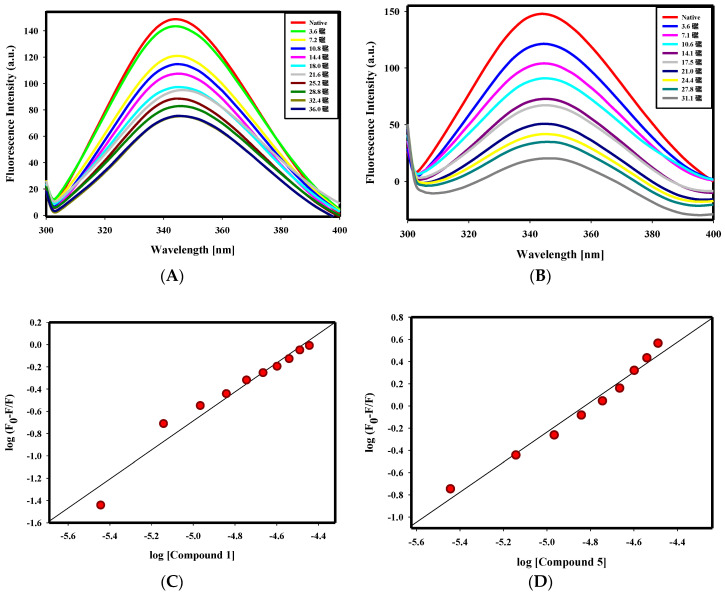
Binding studies of Compounds **1** and **5** with SphK1. Fluorescence emission spectra of SphK1 with increasing concentrations of (**A**) compound **1** (0–36 μM) and (**B**) compound **5** (0–31.1 μM). SphK1 was excited at 280 nm, and emission spectra were recorded in the range of 300–400 nm. Modified Stern–Volmer plot showing quenching of SphK1 fluorescence with increasing concentration of (**C**) Compound **1** and (**D**) Compound **5**.

**Figure 5:**
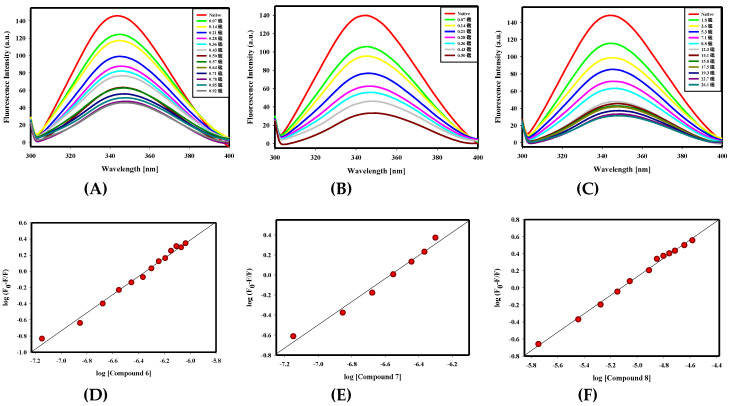
Binding studies of Compounds **6**, **7**, and **8** with SphK1. Fluorescence emission spectra representing SphK1 with the addition of (**A**) Compound **6** (0–0.92 μM), (**B**) Compound **7** (0–0.5 μM), and (**C**) Compound **8** (0–26.1 μM). SphK1 was excited at 280 nm, and emission spectra were recorded in the range of 300–400 nm. Modified Stern–Volmer plot showing quenching of SphK1 fluorescence with increasing concentrations of (**D**) Compound **6**, (**E**) Compound **7**, and (**F**) Compound **8**.

A fluorescence quenching experiment was conducted to measure the binding affinity of designed inhibitors with SphK1 [59]. Different stock concentrations of the compounds were prepared for fluorescence experiments. The protein was excited at 280 nm, and emission spectra were recorded in the range of 300–400 nm. Binding affinities of all the compounds with SphK1 were determined by observing changes in the fluorescence intensity with increasing concentrations of compounds until the saturation point achieved. A significant decrease in the fluorescence intensity of SphK1 upon the addition of specific compounds, indicating the formation of a stable protein-ligand complex. In particular, Compounds **1**, **5** (Figure 4), and **8** (Figure 5C) shown excellent binding affinities for SphK1. Compounds **6** and **7** also showed significantly high binding for SphK1 at very low concentrations (Figure 5). Compounds **2**, **3**, and **4** displayed a moderate binding affinity with SphK1 (Figure S2). There was no significant quenching observed for Compounds **10** (Figure S3B) and **11** (Figure S3C) even at high concentrations. However, Compound **9** (Figure S3A) showed weak binding to the SphK1.

The modified Stern–Volmer plots (Figure 4C,D and Figure 5D–F; Figures S2D–F and S3D) were analyzed to estimate the binding constant (*K*_a_) and the number of binding sites per SphK1 molecule (*n*). Table 1 shows the binding parameters of all compounds under investigation with SphK1. We observed that Compounds **1**, **5**, **6**, **7**, and **8** possess considerable binding affinities with *K*_a_ values in the range of 10^5^–10^7^ M^−1^ with the single binding site (Table 1). These compounds were selected for further studies to measure other properties towards therapeutic implications.

#### 2.2.2. Isothermal Titration Calorimetry

**Figure 6:**
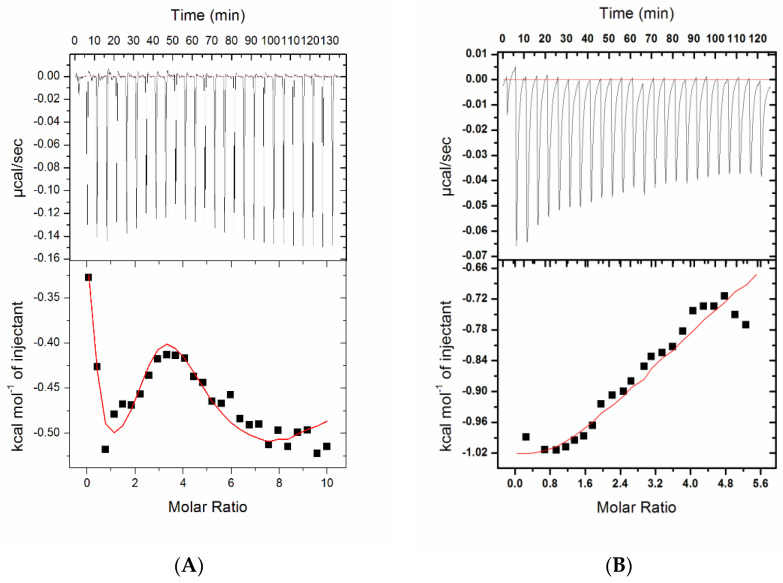
ITC experiment showing the titration of SphK1 with (**A**) Compound **1** and (**B**) Compound **7**. The upper panel depicts the raw data for heat produced against time for each titration of SphK1 with ligands. The bottom panel shows the binding isotherm formed by the integration of peak area and its normalization to yield a plot of molar enthalpy change against Compounds **1**/**7** and SphK1 ratio. The red color line represents the fitted curve.

The binding affinities and the nature of the interactions of compounds that showed high binding affinities in the fluorescence experiments (**1**, **5**, and **7**) with SphK1 were further probed with the help of ITC. During the ITC experiments, 15 μM of SphK1 was titrated with increasing concentrations of Compounds **1**, **5**, and **7** at 25 °C. ITC of Compound **5** with SphK1 resulted in precipitation of the ligand (data not shown). Figure 6 presents the ITC results with the upper panel representing the experimental raw data. The exothermic binding reaction for Compound **1** (Figure 6A) and Compound **7** (Figure 6B) with SphK1 were signified by negative pulses of heat generated. The bottom panel denotes the integrated heat pulses to time as a function of the molar ratio of [Compound **1**]/[SphK1] (Figure 6A) and [Compound **7**]/[SphK1] (Figure 6B) during the binding reaction and concomitant complex formation. The binding isotherm curve represents total heat exchanged per injection during SphK1 interaction with the respective compounds. The heat-related with the corresponding compounds (**1** and **7**) and buffer interactions were also experimentally determined and deducted from the changes in the heat involved during titrations with SphK1. Thermodynamic parameters were analyzed from the pattern of binding isotherms obtained for both compounds with SphK1 by fitting it with two (Compound **1**) and four (Compound **7**) binding site models by using in-built functions of origin software. The *K*_D_ value was in the micromolar range, suggesting a stronger binding affinity of Compounds **1** and **7** towards SphK1, and favorable interactions between these partners were revealed by the negative value of Gibbs free energy (Table 2). The overall free energy of binding was negative for the interaction of Compound **7** with SphK1, indicating that the process could be considered as highly entropy-driven. This is likely to compensate for the positive value of enthalpy and, in turn, could be due to various factors such as the size of the ligand molecule [60,61].

#### 2.2.3. Enzyme Inhibition Assay

**Figure 7:**
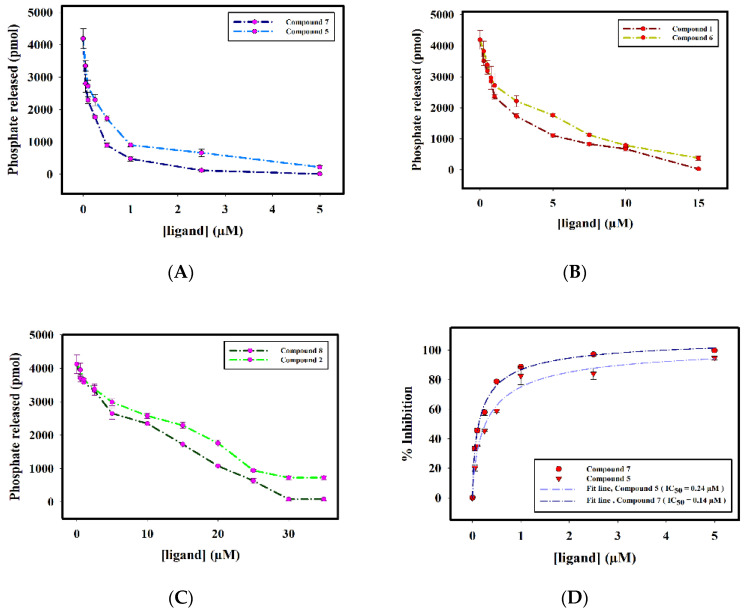

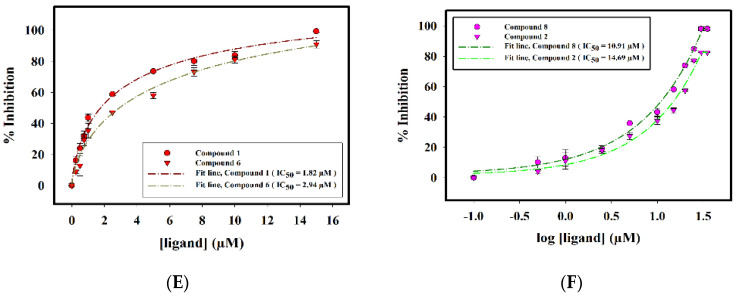
Inhibition of ATPase activity of SphK1. The amount of phosphate released from the hydrolysis of ATP was measured using the standard phosphate curve. Dose-response curve depicting the effect of increasing concentrations of (**A**) Compounds **5** and **7** (0–5 μM), (**B**) Compounds **1** and **6** (0–15 μM), and (**C**) Compounds **8** and **2** (0–30 μM) on the ATPase activity of SphK1. Plots (**D** to **F**) represent percent inhibition in ATPase activity of SphK1 as a function of increasing concentrations of (**D**) Compounds **5** and **7**, (**E**) Compounds **1** and **6**, and (**F**) Compounds **8** and **2**. The IC_50_ value was calculated by fitting the curve obtained from two independent experiments.

Enzyme inhibitory potentials of all the synthesized compounds towards SphK1 were determined by malachite green ATPase inhibition assays. During the initial screening, the maximum concentration of all compounds (50 μM) was used, which revealed that Compounds **1**, **2**, **5**, **6**, **7**, and **8** inhibited SphK1 activity while **3**, **4**, **9**, **10**, and **11** did not show any considerable inhibition (Figure S4). To further evaluate the inhibitory potential and to calculate the IC_50_ values of the compounds’ hits revealed from initial screening, an enzymatic assay of SphK1 was performed across a narrower range (0–35 μM). The ATPase activity of SphK1 was quantified in terms of hydrolyzed phosphate in picomolar concentration using a phosphate standard curve as described [62,63]. The decrease in the activity of SphK1 was plotted in terms of percentage inhibition against an increasing dose of respective compounds, which revealed IC_50_ values in the micromolar range (Figure 7, Table 3). We observed that Compounds **5** and **7** effectively inhibited SphK1 with very low IC_50_ values of 0.24 ± 0.02 μM and 0.14 ± 0.01 μM, respectively (Figure 7D), while Compounds **8** and **2** inhibited SphK1 activity with moderately high IC_50_ values of 10.91 ± 0.02 μM and 14.69 ± 0.02 μM, respectively (Figure 7F). Surprisingly, Compound **1** showed a better IC_50_ value of 1.82 ± 0.06 μM than compound **6**, which inhibited SphK1 with an IC_50_ value of 2.94 ± 0.07 μM. The enzyme inhibition results were consistent with fluorescence and ITC data and point to Compounds **1**, **5**, **6**, and **7** as effective inhibitors. The attractive binding affinity towards SphK1 could be exploited for further modification to design more potent and selective inhibitors of SphK1.

#### 2.2.4. Interaction of Selected Inhibitors with SphK1

**Figure 8:**
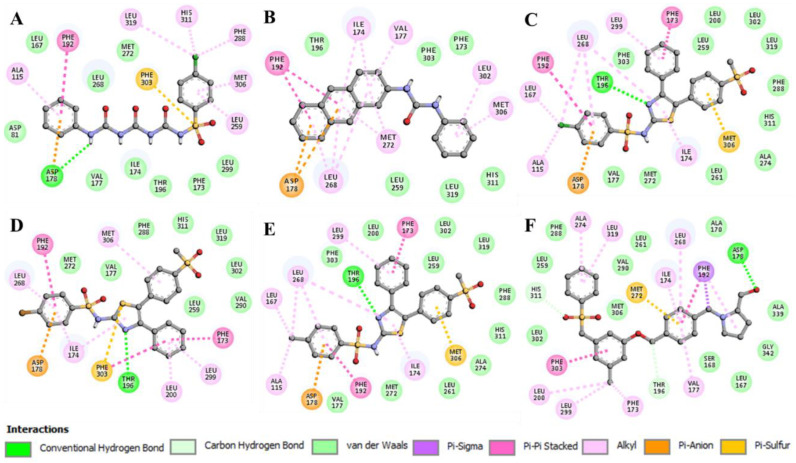
Interactions of selected compounds with SphK1. 2D structural representation of SphK1 residues interacting with (**A**) Compound **1**, (**B**) Compound **5**, (**C**) Compound **6**, (**D**) Compound **7**, (**E**) Compound **8**, and (**F**) PF-543.

**Figure 9:**
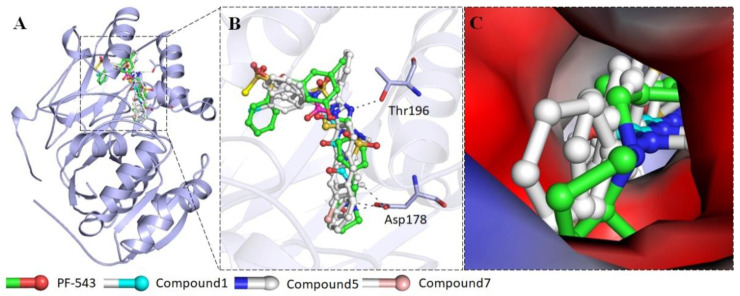
Structural representation of the binding pattern of Compounds **1**, **5**, and **7** along with PF-543 in the substrate-binding site cavity of SphK1. (**A**) Cartoon representation of SphK1 showing ligands bound to the active site cavity. (**B**) Interaction of Compounds **1**, **5**, and **7** to the SphK1 residues. (**C**) Surface model highlighting the active site pocket of SphK1.

All compounds were subjected to docking analysis with SphK1, which predicted binding energy ranging between −5.2 and −10.7 kcal/mol (Table 1). The docking analysis correlated well with our fluorescence binding studies that revealed Compounds **1**, **5**, **6**, **7**, and **8** as possess appreciable binding affinity towards SphK1 (Table 1) and inhibited its ATPase activity in the micromolar range (Table 3). Therefore, Compounds **1**, **5**, **6**, **7**, and **8** were further explored to highlight the potential interactions with active site residues of SphK1 and deduce possible mechanisms of inhibition. It was observed that the complexes of SphK1 and selected compounds were stabilized by several non-covalent interactions offered by the lipid substrate binding site (Asp 178, Thr 196, Ile 174, and Val 177) of SphK1 (Figure 8, Table S1). The interaction patterns for Compounds **1** (Figure 7A) and **5** (Figure 7B) were found to be the same and shared most of the active site residues of SphK1, while Compounds **6** (Figure 8C), **7** (Figure 8D), and **8** (Figure 8E) shared a similar pattern. A common set of interacting residues that were found to be present for all the selected compounds were Asp 178, Thr 196, Met 272, Phe 192, Leu 268, Val 177, Phe 173, Leu 319, His 311, Leu 259, and Phe 303. Interestingly, Compounds **5**, **6**, **7**, and **8** had π-anion interaction with Asp178, except Compound **1**. Figure 9 depicts the binding pattern of Compounds **1**, **5**, and **7** along with PF-543 towards SphK1. The residues involved in interactions with SphK1 and the molecules are noteworthy as these block the accessibility of sphingosine and ATP in the binding cavity of SphK1. We observed that all three Compounds **1**, **5**, and **7** interacted with the substrate-binding site of SphK1 and mimicked the pose of a known co-crystallized SphK1 inhibitor PF-543 (Figure 9B). All three compounds along with PF-543 bound within the deep cavity of the SphK1 binding pocket (Figure 9C). After analyzing the interaction patterns, we conclude that Compounds **1**, **5**, and **7** might serve as lipid substrate competitive inhibitors of SphK1, as all participated in significant non-covalent interactions with Asp178, Val177, Ile174, Phe192, and Thr196, which are present at the substrate-binding site of SphK1.

## 3. Discussion

SphK1/S1P promotes fundamental cellular processes including cell survival, proliferation, migration, and immune function [64]. Several pathological conditions including cancer, diabetes, inflammatory, and neurodegenerative diseases have been linked to defective homeostatic levels of SphK1/S1P [65]. Thus, there is an urgent need for the development of effective and specific inhibitors of SphK1 for the pharmacologic intervention of these diseases. Despite many reports of potential SphK1 inhibitors, the lack of specificity and lower potency poses major challenges for anti-SphK1 drug design [65]. A well-known drug molecule from previous studies, viz. PF-543, despite being highly selective for SphK1, lacked the desired cytotoxicity against cancer cells at pharmaceutically relevant concentrations [37,66,67].

Given the major challenges involved in the designing of potent inhibitors of SphK1, the current study undertaken is a preliminary step towards the identification of different scaffolds that could prove to be useful for the development of selective and potent inhibitors of SphK1. 2-Aminothiazole [45,51] phenyl sulfoxide derivatives [54] and guanidine derivatives [40] have been reported as important pharmacophores enabling inhibition of SphK1. In the present work, a set of 11 novel small molecules (Figure 3) have been synthesized and screened for their inhibitory potential against SphK1. Our small molecules represent a diverse set of scaffolds including the novel diarylsulfonyltriurea (Compounds **1** and **2**), benzothiazole-based sulfonylurea and sulfonamide (Compounds **3** and **4**), anthracene-based urea (Compound **5**), diaryl-substituted thiazole-based sulfonamide (Compounds **6**, **7**, and **8**) and amino acid-based urea and N-monosubstituted urea derivatives (Compounds **9**, **10**, and **11**). Initially, all the compounds were docked with SphK1, which predicted compounds from **1** to **8** with a high binding affinity (Table 1).

The fluorescence binding studies correlated well with docking analysis. We found that Compounds **7**, **6**, **5**, **1**, **8**, **3**, **2**, **4**, and **9** binds to SphK1 in order of decreasing the *K*_a_ value (Table 1). Among these compounds, we observed that Compounds **7**, **6**, **5**, and **1** bound to the SphK1 with a higher affinity. The binding parameters calculated from ITC measurements further suggested a strong interaction of both Compounds **7** and **1** to SphK1 (Figure 6 and Table 2). We further examined the ATPase activity of SphK1 with increasing concentration of Compounds (**1**, **2**, **5**, **6**, **7**, and **8**), which revealed IC_50_ values in the micromolar range (Table 3). The enzyme inhibition assay further complemented our fluorescence and ITC results.

In-depth, molecular docking of Compounds **1**, **5**, **6**, **7**, and **8** with SphK1 examined the binding pattern and identified the molecular interactions (Figure 8). The interaction analysis suggested that Compounds **1**, **5**, and **7** occupied the sphingosine binding site of SphK1 (Figure 9). These results suggested that Compounds **1**, **5**, and **7** might decrease the substrate accessibility of SphK1 by acting as a competitive inhibitor that ultimately leads to enzyme inhibition. Interestingly, inhibitory activity towards SphK1 varied dramatically with substitutions within the same parent structure. This distinction was especially evident between Compounds **1** and **2**, as also between **6**, **7**, and **8**. While urea and sulfonylurea derivatives have been reported as SphK1 inhibitors, this is the first report of a sulfonyltriurea providing the scope of SphK1 inhibition. These results are promising for a deeper examination of the corresponding scaffolds and elucidation of factors that assist in inhibitory activity. Overall, our results imply that Compounds **1**, **5**, **6**, and **7** could be used as novel scaffolds for the generation of lead molecules that could be further optimized for the future development of potent and selective SphK1 inhibitors.

## 4. Materials and Methods

### 4.1. Chemistry

#### 4.1.1. Materials

Chemicals were procured from Sigma Aldrich, Merck, or Alfa-Aesar. Solvents were obtained from Merck Chemicals, SD Fine Chem Limited, or Finar Limited and were used without further purification. Nuclear magnetic resonance (NMR) spectra were recorded on a 500 MHz Bruker Instrument. Mass spectra were measured by LC-MS on a Waters SYNAPT-G2S-S using electrospray ionization. Chemical shifts were measured in ppm (δ) relative to TMS (0.00 ppm). Coupling constants (*J*) are reported in Hertz (Hz). Silica gel plates were used for TLC analysis.

#### 4.1.2. Synthesis

1. 1. General procedure for the synthesis of sulfonyltriurea (Compounds **1**, **2**, and **3**)

Compounds **1**, **2**, and **3** were synthesized according to the reported method [68]. Sulfonyl chloride (2.4 mmol) was stirred in pyridine (5.0 mmol) for 5 min. The resultant solution was added to a mixture of sodium cyanate (3.9 mmol) in acetonitrile (10 mL) and allowed to stir at room temperature. After 4 h, aniline derivatives (4.4 mmol) were added to the reaction mixture and stirred for 1 h at room temperature. The resulting reaction mixture was poured on crushed ice-cold water and acidified with dilute HCl (pH 5–6). The aqueous layer was extracted with ethyl acetate and was washed with brine and dried over anhydrous sodium sulphate (Na_2_SO_4_). The ethyl acetate layer was concentrated under reduced pressure, and the solid residue was purified by flash column chromatography.

2. General procedure for the synthesis of sulfonamide derivatives (Compound **4**)

Compound **4** was synthesized according to the reported method [69]. Sulfonyl chloride (1 equiv.) was added to a solution of amine (1 equiv.) in dry pyridine. The reaction mixture was heated at 100 °C for 1–1.5 h. After completion, the reaction mixture was cooled, poured into ice-cold water, and acidified with dilute HCl. The solid product was collected by filtration and purified by column chromatography to afford the desired compound.

3. General procedure for the synthesis of Compound **5**

Phenyl isocyanate (2 mmol) was added to 2-aminoanthracene (2 mmol) in dry DCM (10 mL) under a nitrogen atmosphere and stirred continuously at room temperature for 4 h. The reaction mixture was filtered and washed with dry DCM to get a pure desire compound.

4. General procedure for the synthesis of Compounds **6**, **7**, and **8**

**Scheme 1:**
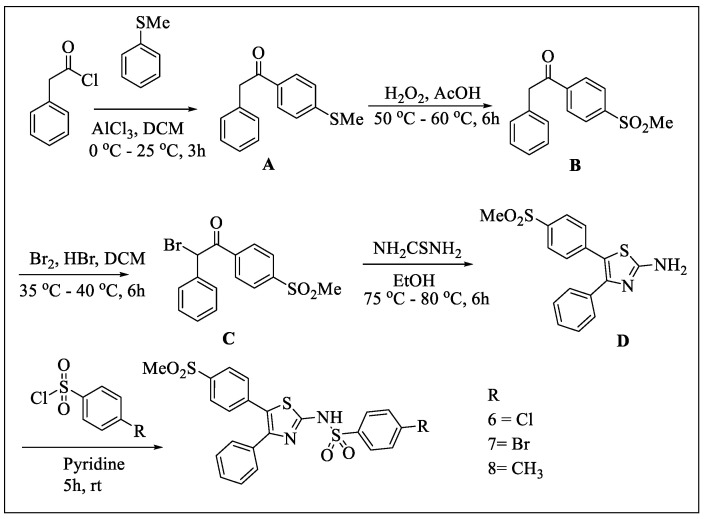
Synthesis of Compounds **6**, **7**, and **8**.

Compounds **6**, **7**, and **8** were synthesized according to Scheme 1. Friedel Craft acylation of phenyl acetyl chloride with thioanisole in the presence of aluminum chloride (AlCl_3_) in dry dichloromethane (DCM) led to the formation of compound A [70], which after oxidation by hydrogen peroxide (H_2_O_2_) in the presence of acetic acid gave compound B [70]. Compound B was treated with bromine in acetic acid in the presence of hydrobromic acid (HBr) as a catalyst to obtain compound C. Reaction of C with thiourea under Hanztch’s thiazole condensation condition gave intermediate compound D [71]. Suitable sulfonyl chlorides (1.1 mmol) were added to a solution of compound D (0.6 mmol) in pyridine (4–6 mL), and the resultant mixture was stirred for 5 h at room temperature. The reaction mixture was then poured on crushed ice and acidified with dilute HCl (pH 5–6). The resulting solid was filtered and further purified using flash chromatography.

5. General procedure for the synthesis of Compound **9**

Phenyl isocyanate (1 equiv.) was added to a mixture of amino acid ester hydrochloride salt (1 equiv.) and DIPEA (3.5 equiv.) in dry DCM and allowed to stir for 90 min at room temperature under nitrogen atmosphere. After completion of the reaction, the reaction mixture was poured on crushed ice and acidified with dilute HCL (pH 5–6). The aqueous layer was extracted with ethyl acetate, and the organic layer was washed with brine and dried over anhydrous sodium sulphate (Na_2_SO_4_). Ethyl acetate was concentrated under reduced pressure to obtain a solid residue, which was finally purified by flash column chromatography to obtain the desired pure compound.

6. General procedure for the synthesis of Compounds **10** and **11**

A mixture of amino acid ester hydrochloride salt (4 mmol), sodium cyanate (6 mmol), and pyridine (4 mmol) in 5–10 mL of acetonitrile was stirred for 90 min at room temperature. Product formation was monitored by TLC. After completion of the reaction, the reaction mixture was poured over ice-cold water, acidified with dilute HCl (pH 5–6), extracted with ethyl acetate, dried over anhydrous sodium sulphate (Na_2_SO_4_), and concentrated under reduced pressure. Finally, the solid residue was purified by column chromatography to obtain the desired compounds.

### 4.2. Biological Studies

#### 4.2.1. Materials

Luria broth and Luria agar were obtained from HiMedia Laboratories (Mumbai, India). Plasmid pET28b+, DH5α, and BL21-Gold cells were purchased from Qiagen (Hilden, Germany). Ni-NTA column was purchased from GE Healthcare (GE Healthcare Life Sciences, Uppsala, Sweden). N-Lauroyl sarcosine, Tris buffer, DMSO, and other reagents were purchased from Sigma Aldrich (St. Louis, MO, USA). BIOMOL^®^ was obtained from Enzo Life Sciences (Farmingdale, NY, USA). All the reagents used for buffer preparation were of analytical grade.

#### 4.2.2. Expression and Purification of SphK1

Plasmid pET28b+ containing the *SphK1* gene insert was expressed in BL21 Gold cells and subsequently purified by Ni-NTA affinity chromatography as described in our previous communication [72]. In short, 1% of overnight-grown culture was used as an inoculum for secondary culture development. The expression of SphK1 was induced by 1 mM IPTG at optimal cell density (A_600_ nm~0.6) for 3–4 h. Afterwards, harvested culture was pelleted followed by resuspension in lysis buffer for preparation of the inclusion bodies. Finally, inclusion bodies were solubilized in the solubilization buffer (0.5% sarcosine prepared in 50 mM Tris, 150 mM NaCl buffer at pH 8.0), and the supernatant was loaded to the equilibrated Ni-NTA column. His-tagged SphK1 was eluted with 250 mM imidazole, and the eluted fraction was analyzed by SDS-PAGE to determine the purity. The purified protein was later dialyzed for 24 h to get refolded protein, and the concentration was calculated using a molar absorption coefficient of 48,275 M^−1^cm^−1^ at 280 nm on a UV–visible spectrophotometer (Jasco V-660, Japan).

#### 4.2.3. Fluorescence Binding Studies

The binding studies of synthesized compounds with SphK1 were conducted on a spectrofluorometer (Jasco FP-8500, Japan) at 25 ± 0.1 °C. The exit and entrance slit width was kept at 10 nm. A stock solution of compounds with a varying concentration of 5–30 mM was prepared in DMSO and then diluted to a working concentration, according to the experimental requirement, in 20 mM Tris buffer with 100 mM NaCl (pH 8.0). A fixed concentration of SphK1 (4 μM) was titrated with increasing concentrations of all the synthesized compounds until the saturation point reached. SphK1 was excited at 280 nm, and the emission spectra were collected from 300 to 400 nm. The decrease in the fluorescence intensity of SphK1 with increasing concentrations of compounds was plotted, and the inverse correlation amongst them was utilized as the criteria for evaluating the kinetic parameters using the modified Stern–Volmer equation (Equation (1)) as described [73,74].

(1) $$\log\frac{\left(Fo-F\right)}{F}=\log K_{a}+n\;\log\;[\mathrm{Inhibitor}]$$

where *F*_o_ denotes fluorescence intensity of SphK1 without the compound, and *F* denotes the fluorescence intensity of SphK1 at a specific concentration of compound at λ_max_. *K*_a_ and *n* were, hence, calculated from Equation (1).

#### 4.2.4. Isothermal Titration Calorimetry

The binding affinities of Compounds **1** and **7** with SphK1 were also evaluated by a VP-ITC microcalorimeter from MicroCal, Inc (GE, MicroCal, USA) at 25 °C. Both protein and compounds were diluted in a buffer (20 mM Tris, pH 8.0, and 100 mM NaCl). An equal concentration of DMSO (1.08% *v/v* for 1 and 1.68% *v/v* for 7) was added to the protein solution to minimize the signal-to-noise ratio during ITC experiments. The samples were degassed before loading. The automated titration started with a false injection of respective compounds (2 μL) into the protein cell followed by 10 μL (24 successive) injection with 300 s interval with constant stirring at 308 rpm. For analysis, the heat released during the interaction of the compound with the buffer was also measured to obtain the final heat of dilution during titration of the respective compound with SphK1. The titration data were further analyzed using MicroCal Origin 7.0. The binding isotherm was fitted with a binding site model for determination of association constant (*K*_a_), enthalpy change (Δ*H*), and entropy change (Δ*S*) as described [75,76].

#### 4.2.5. Enzyme Inhibition Assay

The inhibitory effect of these synthesized compounds on SphK1 activity was evaluated with a standard Malachite Green (BIOMOL^®^ GREEN reagent, Enzo Life Sciences) microtiter-plate assay [77]. For the ATPase assay, compounds were incubated with SphK1 (4 μM) for 1 h at 25 °C followed by the addition of freshly prepared cold ATP (200 μM) and 10 mM MgCl_2_. The reaction was incubated for 30 min at 25 °C and then terminated by adding a double volume of BIOMOL^®^ reagent (200 µL). After 10 min, the absorbance of the green-colored complex was recorded at 620 nm using an ELISA reader. The reaction containing no protein was set up to obtain the background reading of inorganic phosphate. The inhibition of SphK1 activity was plotted in terms of percentage using a standard phosphate curve as described [62,78]. The data points were calculated from three independent experiments in triplicate.

#### 4.2.6. Molecular Docking

The atomic coordinates of SphK1 (PDB ID: 4V24) were downloaded from Protein Data Bank, and the amino acid residues were renumbered according to the UniProt sequence (UniProt identifier: Q9NYA1-1). 2D and 3D structures of all synthesized compounds were prepared using ChemBio3D Ultra 12.0. AutoDock Vina [79] was used for the docking to produce the ligand-receptor interaction model. PyMOL [80] and Discovery Studio Visualizer [81] were used for the analysis of structural features of the SphK1-ligand complex to determine important protein-ligand interactions and binding affinity estimation. A description of the docking method has been described in our previous communications [82,83,84].

## 5. Conclusions

In conclusion, this study revealed that Compounds **1**, **5**, **6**, and **7** with a distinct architecture belonging to diverse groups of N-(4,5-diphenylthiazol-2-yl) benzenesulfonamide, anthracene, and sulfonyltriurea, respectively, might be utilized as potential scaffolds for the generation of lead molecules in the development of selective inhibitors against SphK1.

## Figures and Tables

**Table 1:** Binding parameters of all the synthesized compounds with SphK1 obtained from molecular docking and fluorescence binding studies.

S. No.	Compound ID	Structure	Predicted Affinity Δ*G* ^€^ (kcal/mol)	^¥^ Binding Affinity Constant (*K*_a_), M^−1^	^¥^ Number of Binding Sites (*n*)
**1.**	Compound **1**	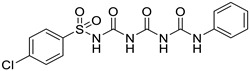	−9.3	6.81 × 10^5^	1.3
**2.**	Compound **2**	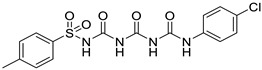	−9.6	2.71 × 10^4^	1
**3.**	Compound **3**	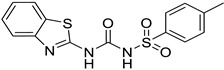	−8.9	3.75 × 10^4^	1
**4.**	Compound **4**	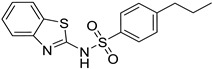	−8.6	1.34 × 10^4^	0.9
**5.**	Compound **5**	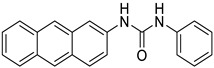	−10.7	1.58 × 10^6^	1.3
**6.**	Compound **6**	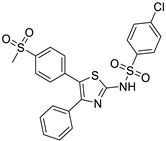	−8.5	1.32 × 10^7^	1.1
**7.**	Compound **7**	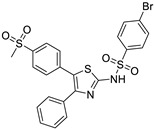	−8.7	3.9 × 10^7^	1.2
**8.**	Compound **8**	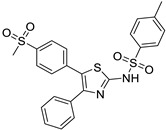	−8.4	3.52 × 10^5^	1
**9.**	Compound **9**	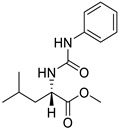	−6.4	9.43 × 10^2^	0.6
**10.**	Compound **10**	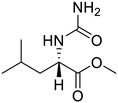	−5.8	NA	NA
**11.**	Compound **11**	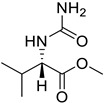	−5.2	NA	NA
**12.**	* PF-543	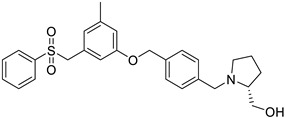	−9.7	NA	NA

^€^ Binding affinity of the synthesized compounds with SphK1 predicted through AutoDock Vina. ^¥^ Binding affinity (*K*_a_) calculated from fluorescence studies. Some of the compounds do not show any fluorescence quenching with SphK1. Hence, the values of K_a_ and n cannot be calculated in such cases and are depicted as not applicable (NA). * PF-543 is used as a reference compound in docking experiments.

**Table 2:** Thermodynamic parameters obtained from calorimetric titrations of Compounds **1** and **7** with SphK1.

Complex	Number of Binding Sites, N	Association Constant *K*_a_, M^−1^	Dissociation Constant *K_D_*, µM	Enthalpy Change Δ*H*, cal/moL	Entropy Change Δ*S*, cal/mol/deg	Δ*G* (Free Energy Change), kcal/mol
Compound **1**	2	*K*_a1_ = 1.63 × 10^4^ ± 9.1 × 10^2^	*K*_D1_ = 61.3	Δ*H*_1_ = −5204 ± 265	Δ*S*_1_ = 1.82	Δ*G*_1_ = −5.75
		*K*_a2_ = 3.67 × 10^3^ ± 3.0 × 10^2^	*K*_D2_ = 272.4	Δ*H_2_* = −1.909 × 10^4^ ± 1.73 × 10^3^	Δ*S*_2_ = −47.7	Δ*G*_2_ = −4.88
Compound **7**	4	*K*_a1_ = 4.79 × 10^4^ ± 3.8 × 10^3^	*K*_D1_ = 20.8	Δ*H*_1_ = −608.7 ± 59.9	Δ*S*_1_ = 19.4	Δ*G*_1_ = −6.39
		*K*_a2_ = 1.81 × 10^5^ ± 6.9 × 10^3^	*K*_D2_ = 5.5	Δ*H_2_* = −934.5 ± 97	Δ*S*_2_ = 20.9	Δ*G*_2_ = −7.16
		*K*_a3_ = 1.19 × 10^4^ ± 4.6 × 10^2^	*K*_D3_ = 84	Δ*H_3_* = 277.1 ± 227	Δ*S*_3_ = 19.6	Δ*G*_3_ = −5.56
		*K*_a4_ = 4.40 × 10^3^ ± 1.2 × 10^2^	*K*_D4_ = 227.3	Δ*H_4_* = −1.898× 10^4^ ± 569	Δ*S*_4_ = −47.0	Δ*G*_4_ = −4.97

**Table 3:** IC_50_ values of the selected compounds for SphK1 inhibition calculated from the ATPase inhibition assay.

S. No.	Compound ID	IC_50_ (μM)
1.	Compound **1**	1.82 ± 0.06
2.	Compound **2**	14.69 ± 0.02
3.	Compound **5**	0.24 ± 0.02
4.	Compound **6**	2.94 ± 0.07
5.	Compound **7**	0.14 ± 0.01
6.	Compound **8**	10.91 ± 0.02

## Supplementary Materials

The following are available online at https://www.mdpi.com/1424-8247/13/6/118/s1. **Figure S1.** Characterization of all synthesized compounds (**1 to 11**) by different spectroscopic techniques like ^1^H NMR, ^13^C NMR, and LC-MS mass spectrometry. **Figure S2.** Binding studies of compound **2, 3 and 4** with SphK1. Fluorescence emission spectra representing SphK1 quenching on the addition of an increasing amount of (**A**) compound **2** (0–47.6 μM), (**B**) compound **3** (0–37.8 μM) and (**C**) compound **4** (0–27.8 μM). SphK1 was excited at 280 nm and emission spectra were recorded in the range of 300–400 nm. Modified Stern-Volmer plot showing quenching of SphK1 fluorescence with increasing concentration of (**D**) compound **2**, (**E**) compound **3** and (**F**) compound **4**. The SV plot was used to calculate binding constant (*K*a) and the number of binding sites (*n*). **Figure S3.** Binding studies of compound **9**,**10** and **11** with SphK1. Fluorescence emission spectra representing SphK1 quenching on the addition of an increasing amount of (**A**) compound **9** (0–26.1 μM), (**B**) compound **10** (0–8.8 μM) and(**C**) compound **11** (0–14.1 μM). SphK1 was excited at 280 nm and emission spectra were recorded in the range of 300–400 nm. (**D**) Modified Stern-Volmer plot showing quenching of SphK1 fluorescence with increasing concentration of compound **9**. The SV plot was used to calculate binding constant (*K*a) and the number of binding sites (*n*). **Figure S4.** Screening of compounds for evaluation of their inhibitory potential against SphK1 by malachite green-based ATPase inhibition assay. The inorganic phosphate released by ATPase activity of SphK1 forms a green complex with the malachite green that absorbs at 620 nm. **Table S1.** List of different non-covalent interactions between compounds and SphK1 interacting residues.
