# Supplementary material for: Design and Development of Novel Urea, Sulfonyltriurea, and Sulfonamide Derivatives as Potential Inhibitors of Sphingosine Kinase 1
Source: Pharmaceuticals (Basel). 2020 Jun 9;13(6):118. doi: 10.3390/ph13060118 (PMC7346089; doi:10.3390/ph13060118)
Supplement: Supplementary file 1 [file pharmaceuticals-13-00118-s001.pdf]

Supplementary Materials

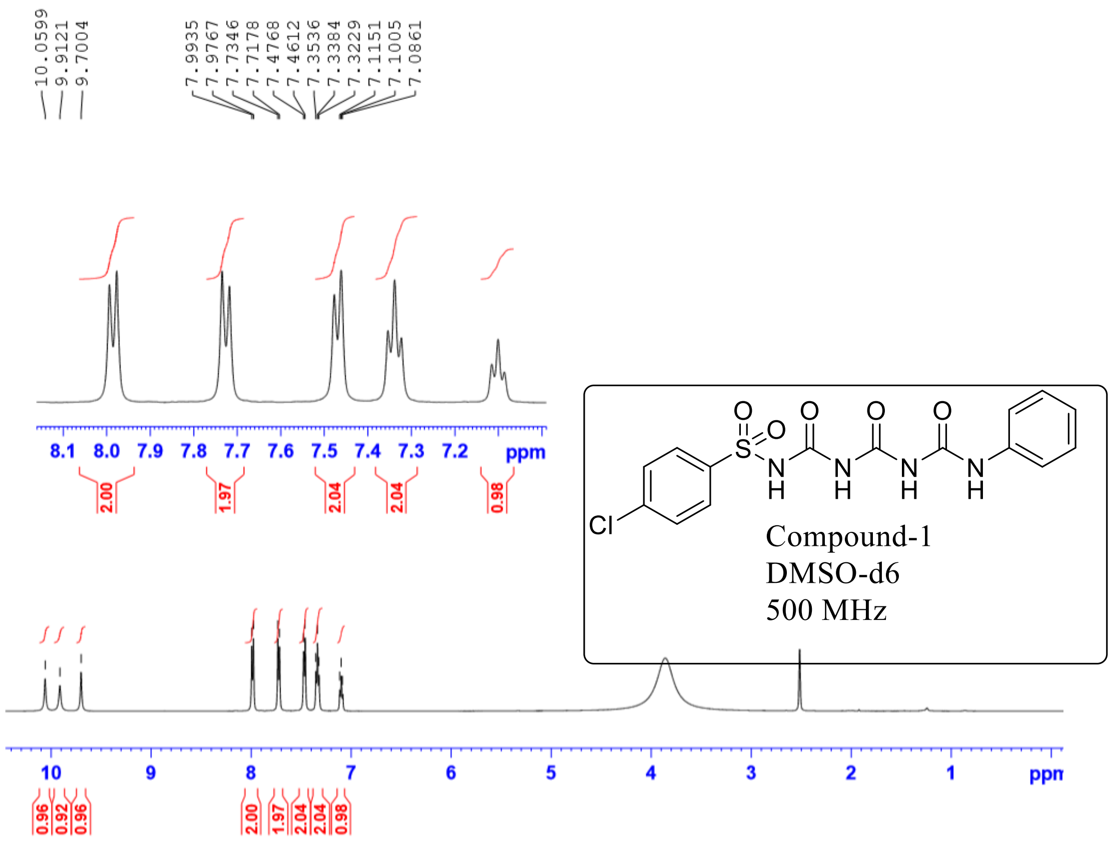

^1H NMR of compound 1

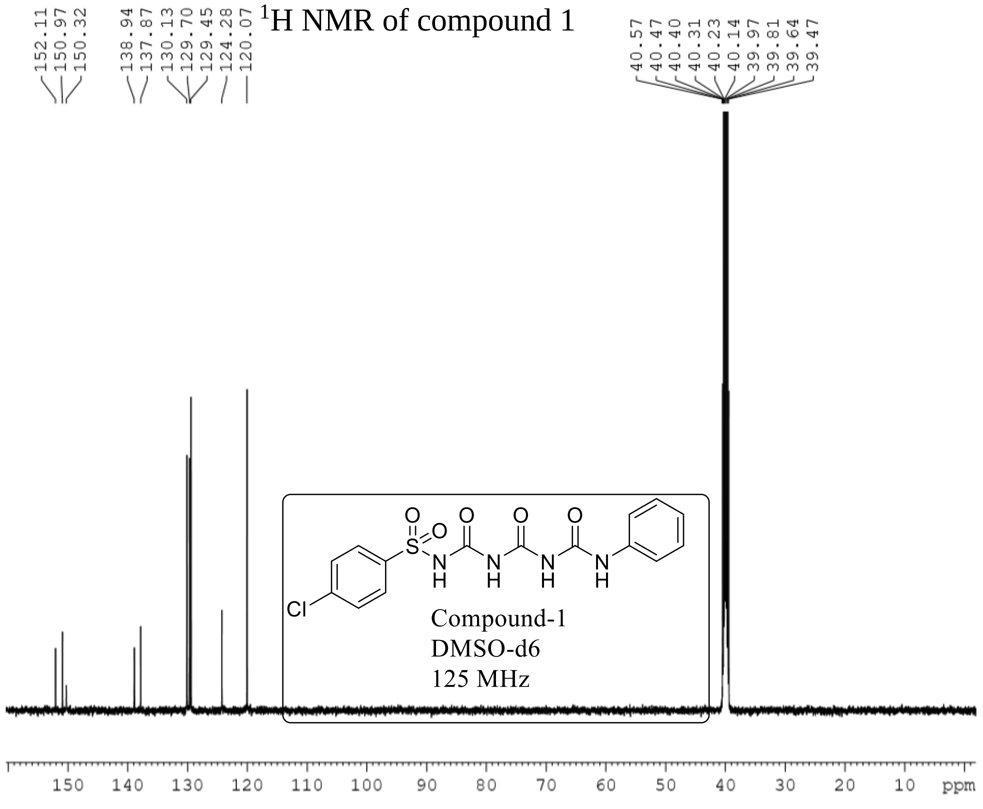

^{13}C NMR of compound 1

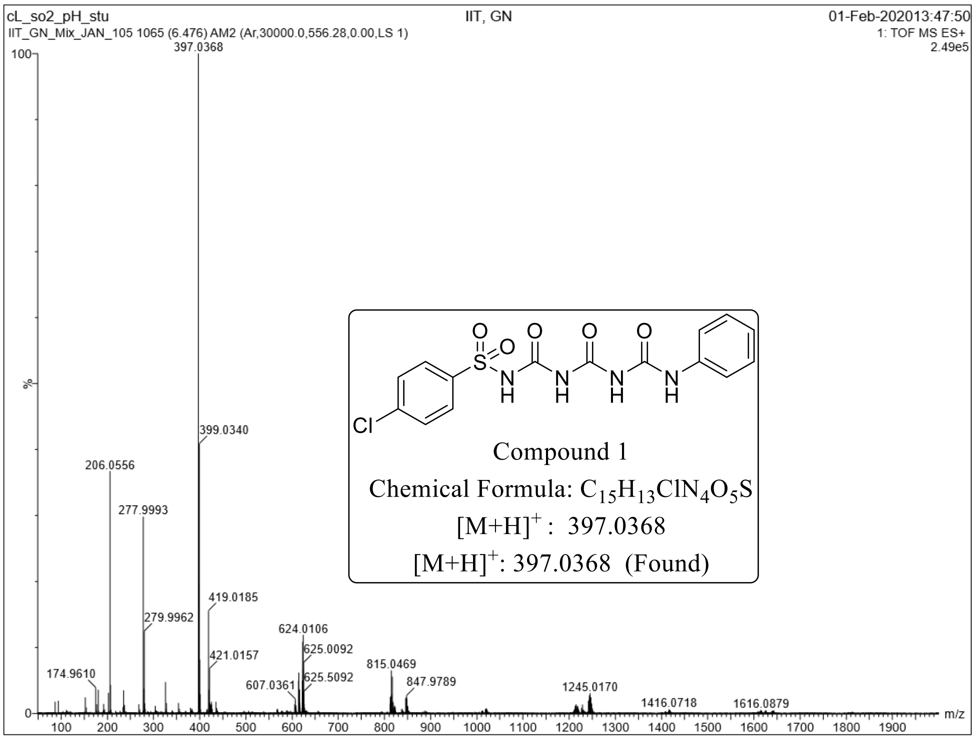

LC-ESIMS of Compound 1

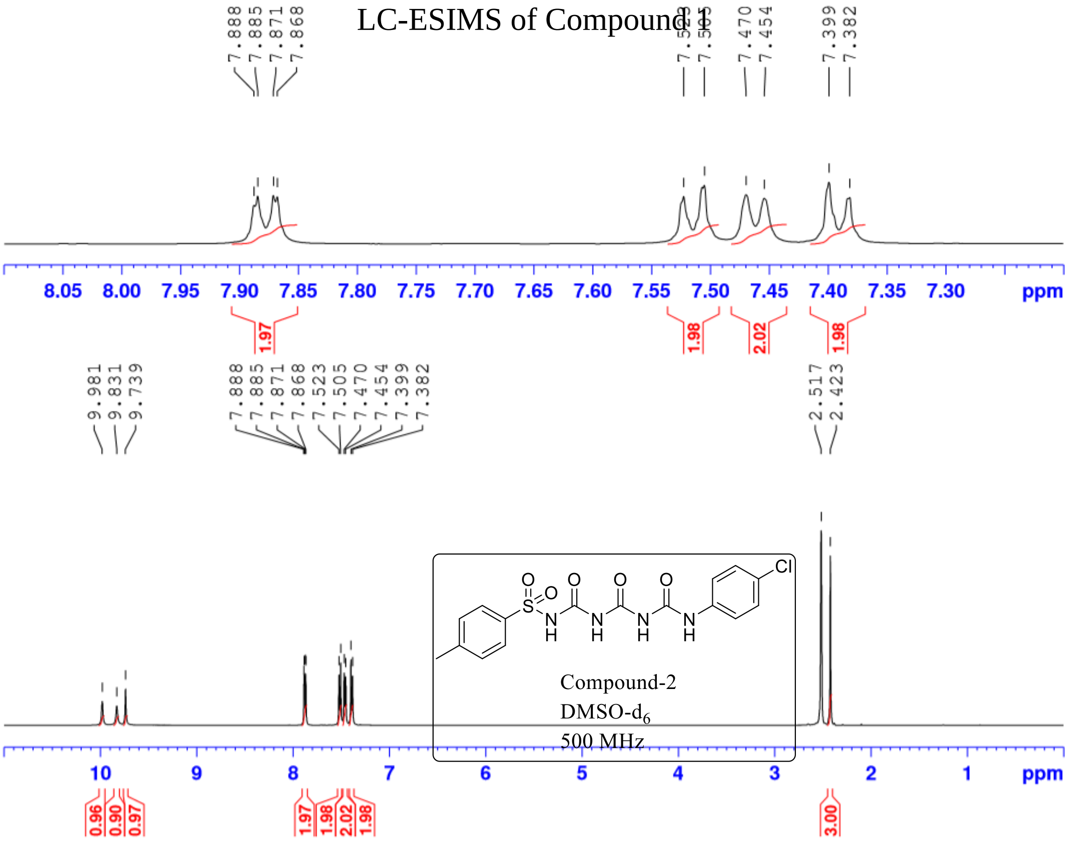

^{1}H NMR of compound 2

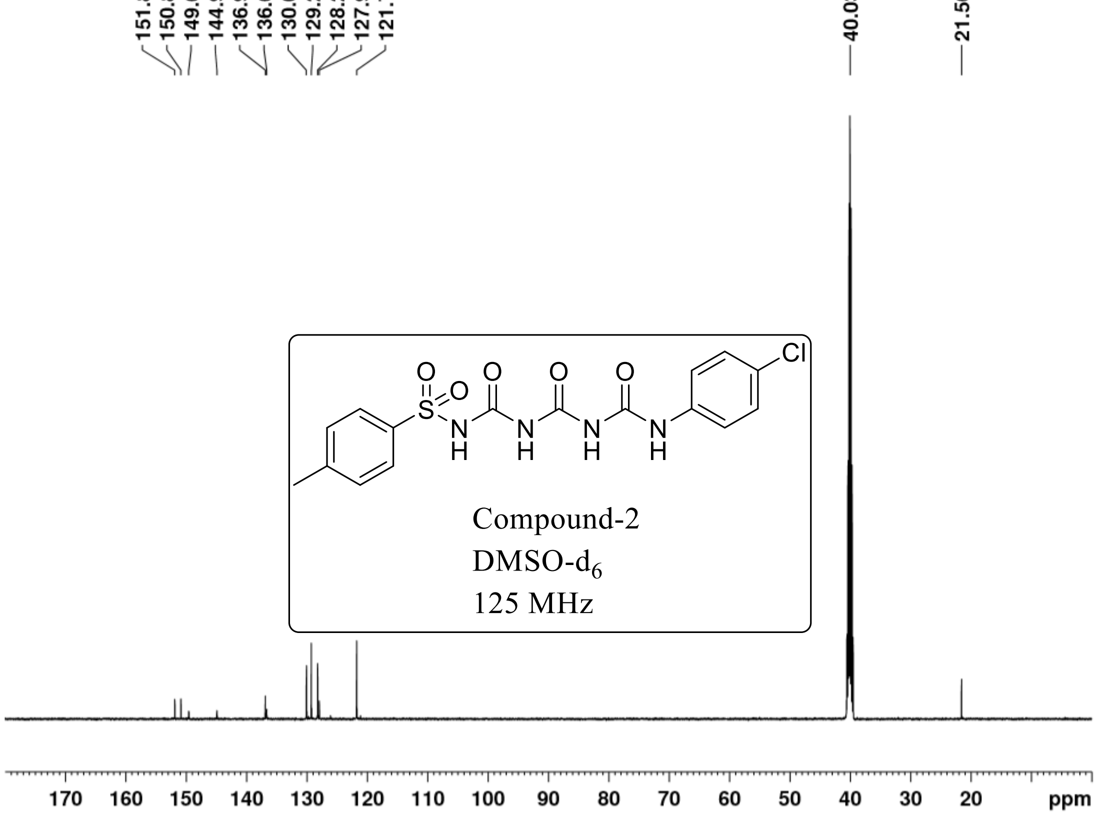

^{13}C NMR of compound 2

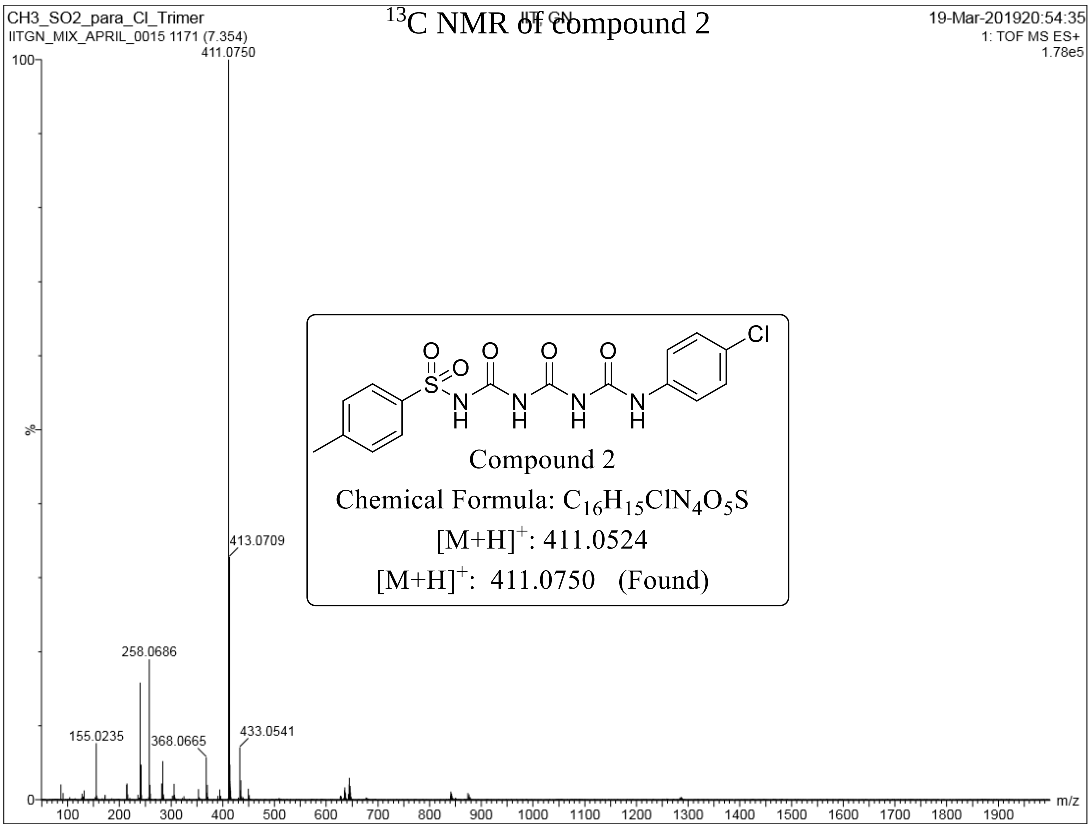

LC-ESIMS of Compound 2

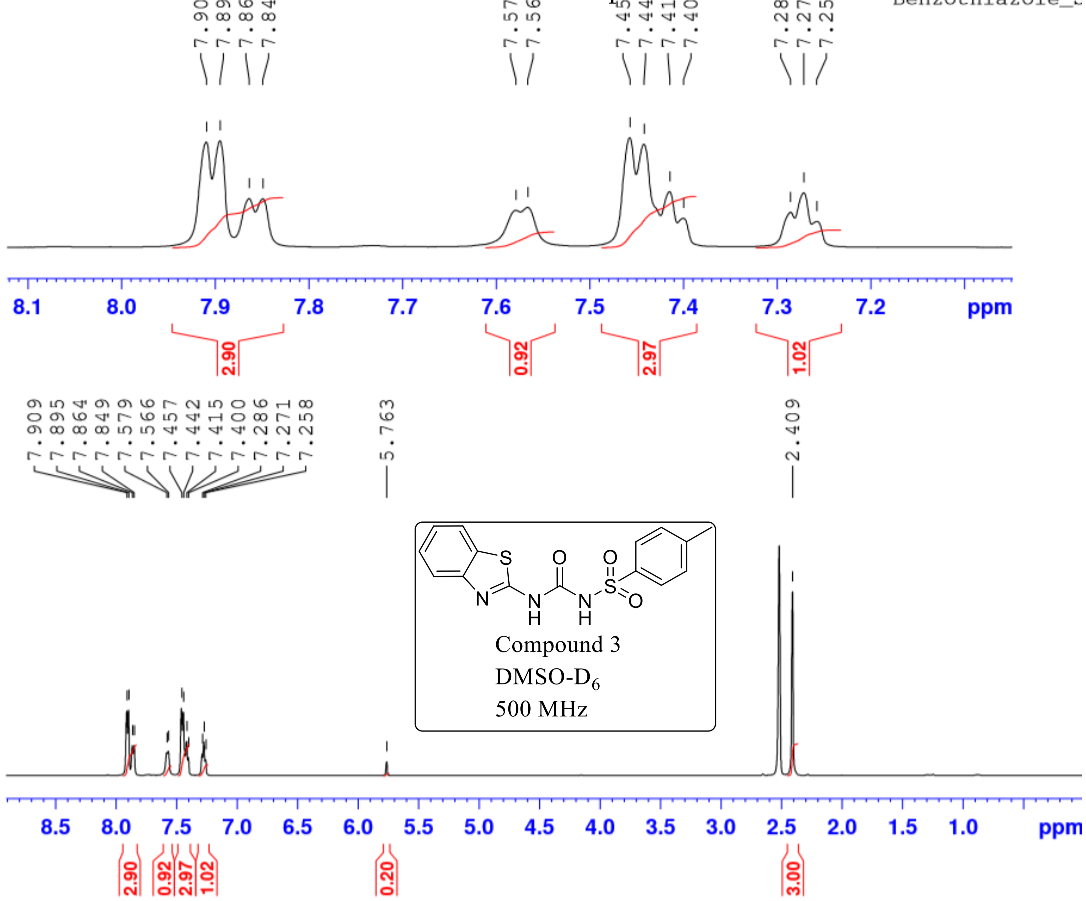

^{1}H NMR of compound 3

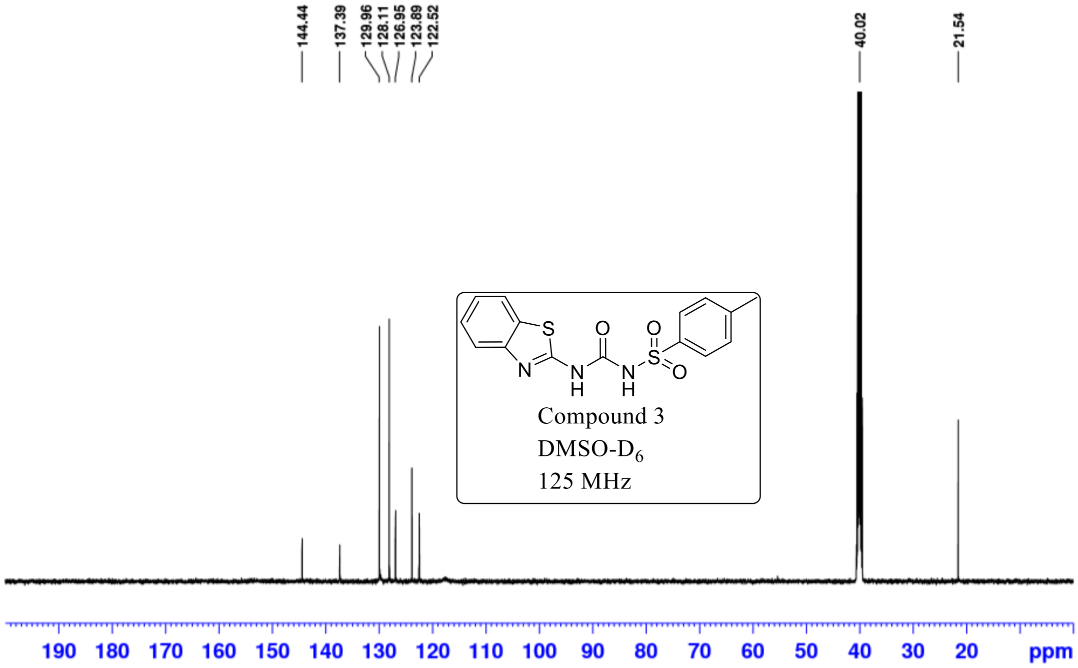

^{13}C NMR of compound 3

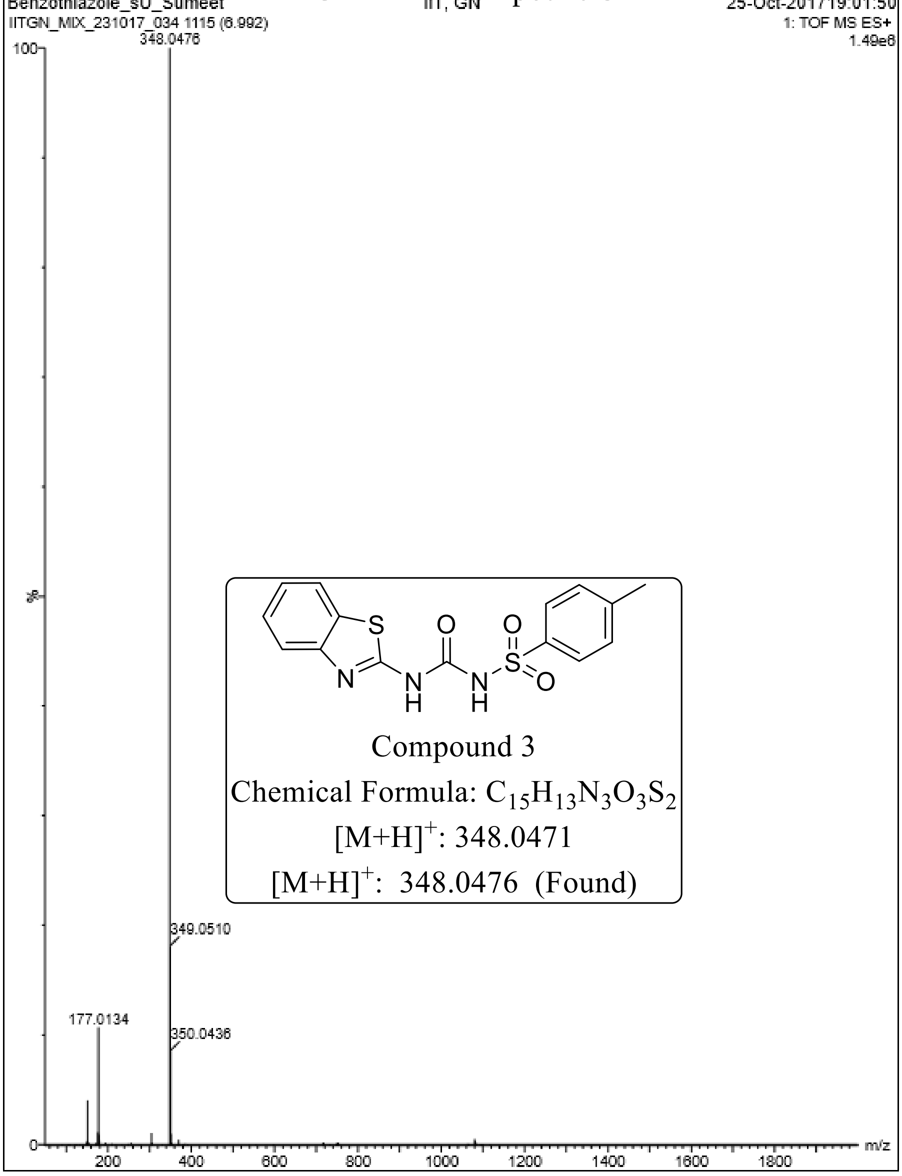

LC-ESIMS of Compound 3

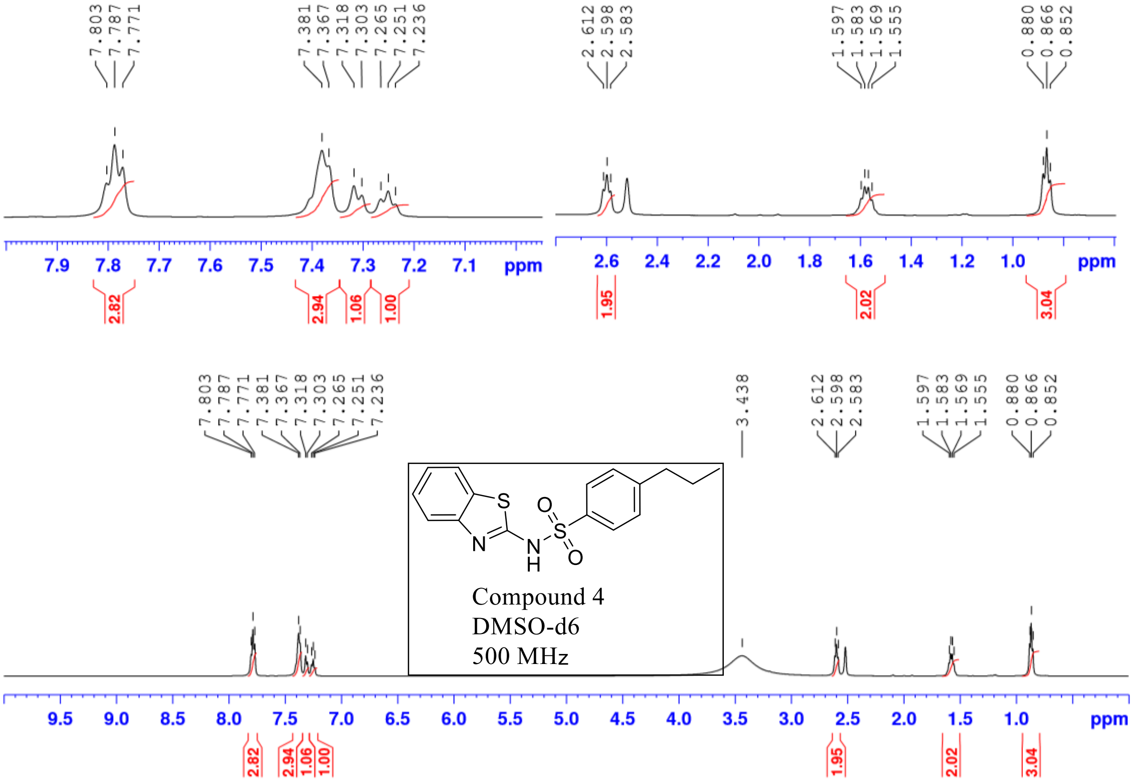

^{1}H NMR of compound 4

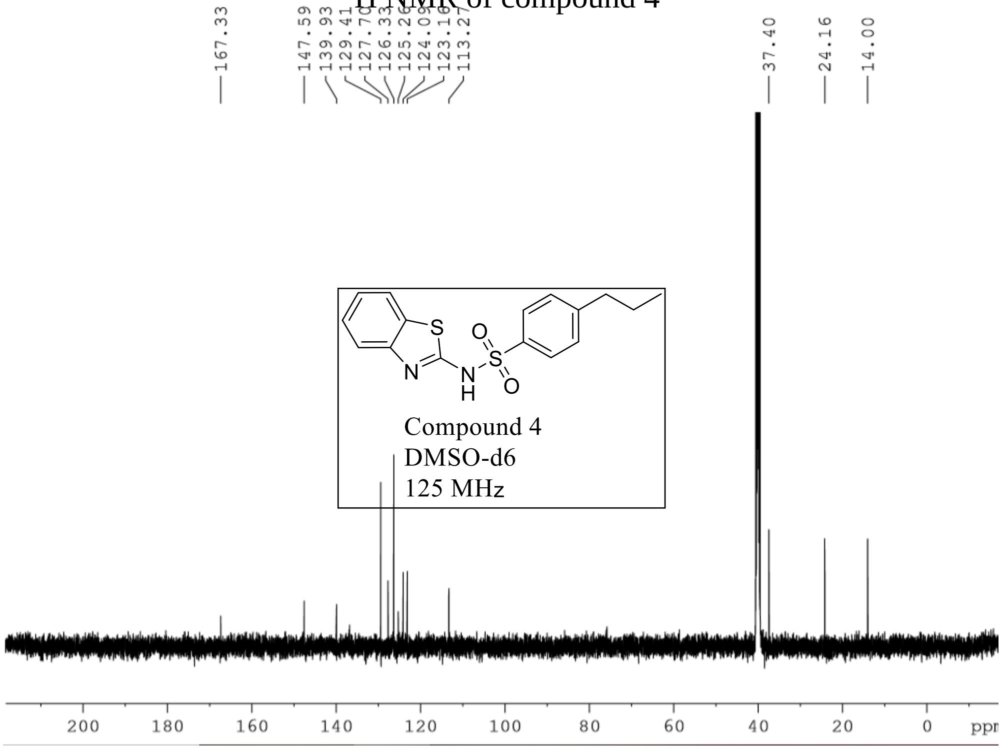

^{13}C NMR of compound 4

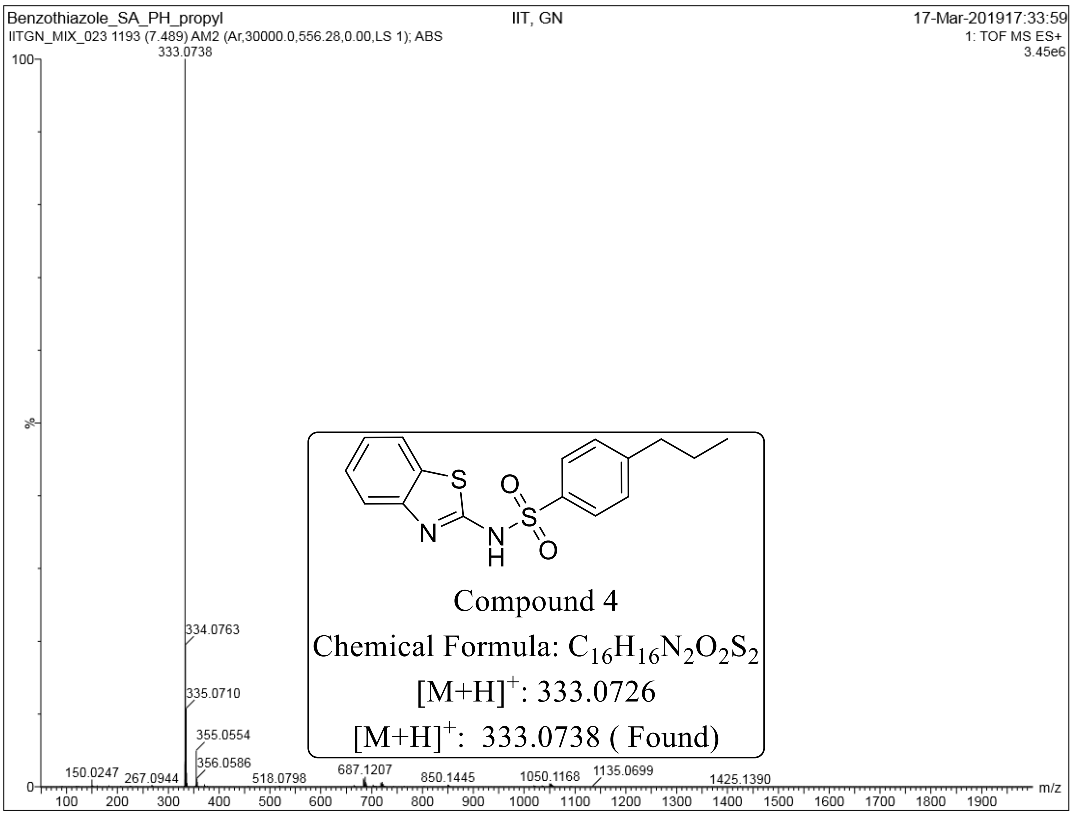

LC-ESIMS of Compound 4

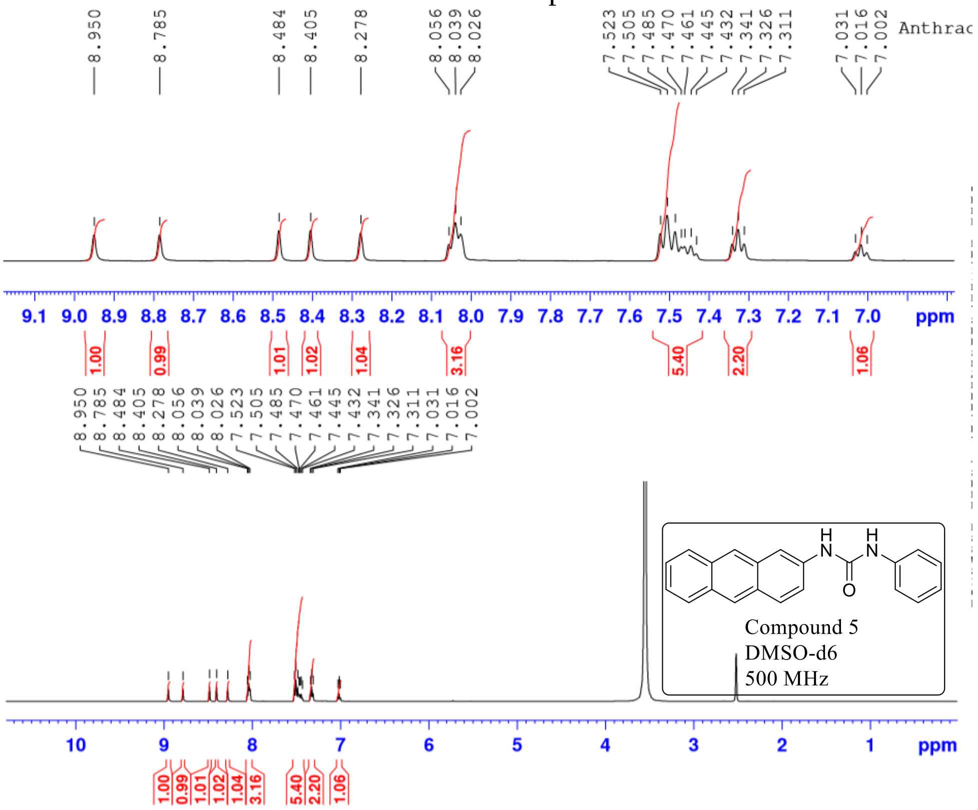

^{1}H NMR of compound 5

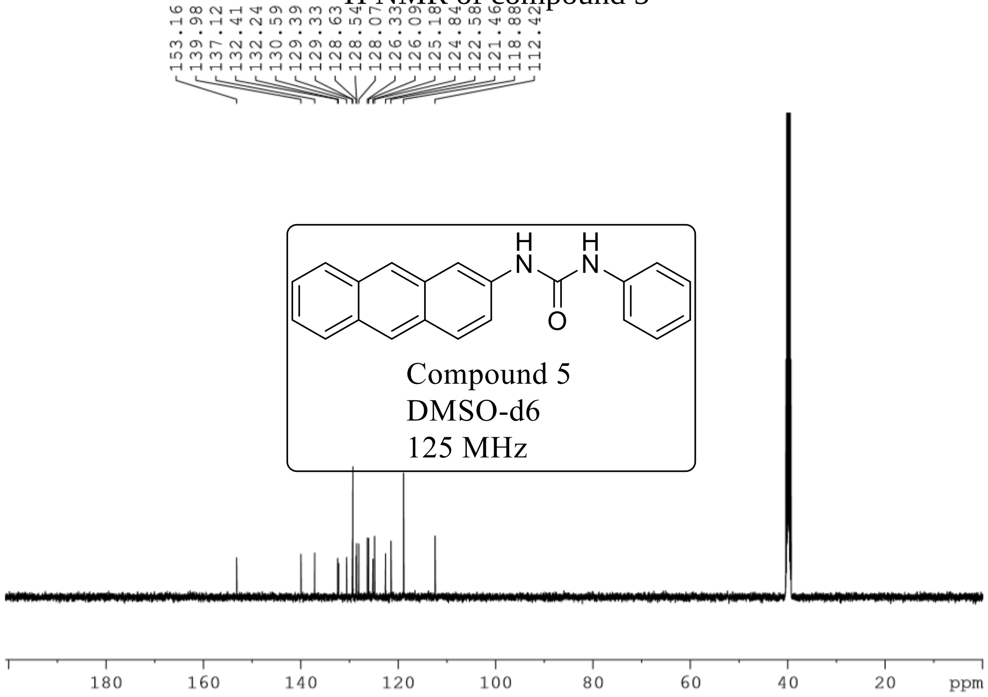

^{13}C NMR of compound 5

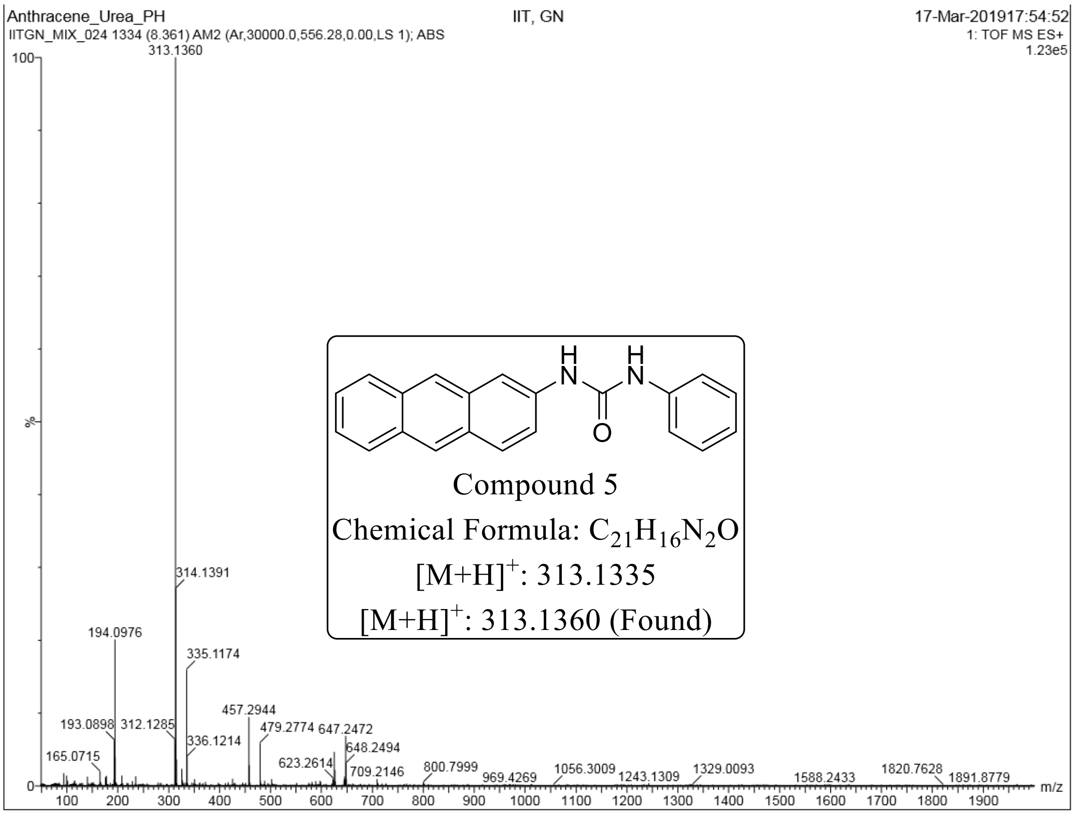

LC-ESIMS of Compound 5

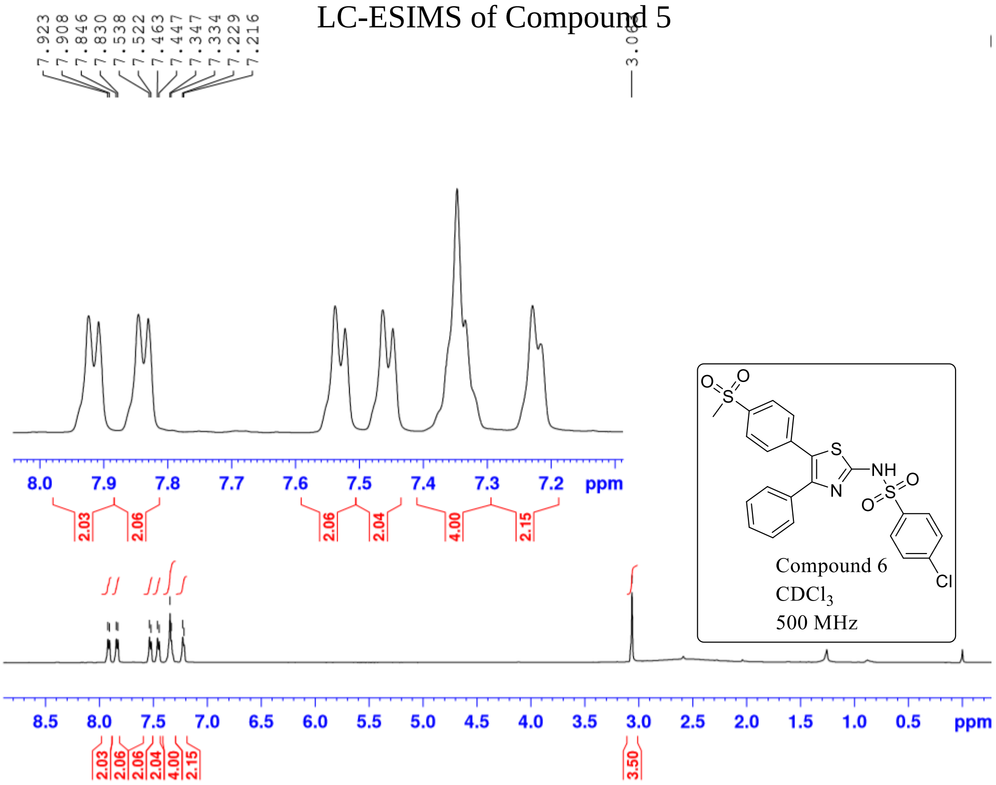

^{1}H NMR of compound 6

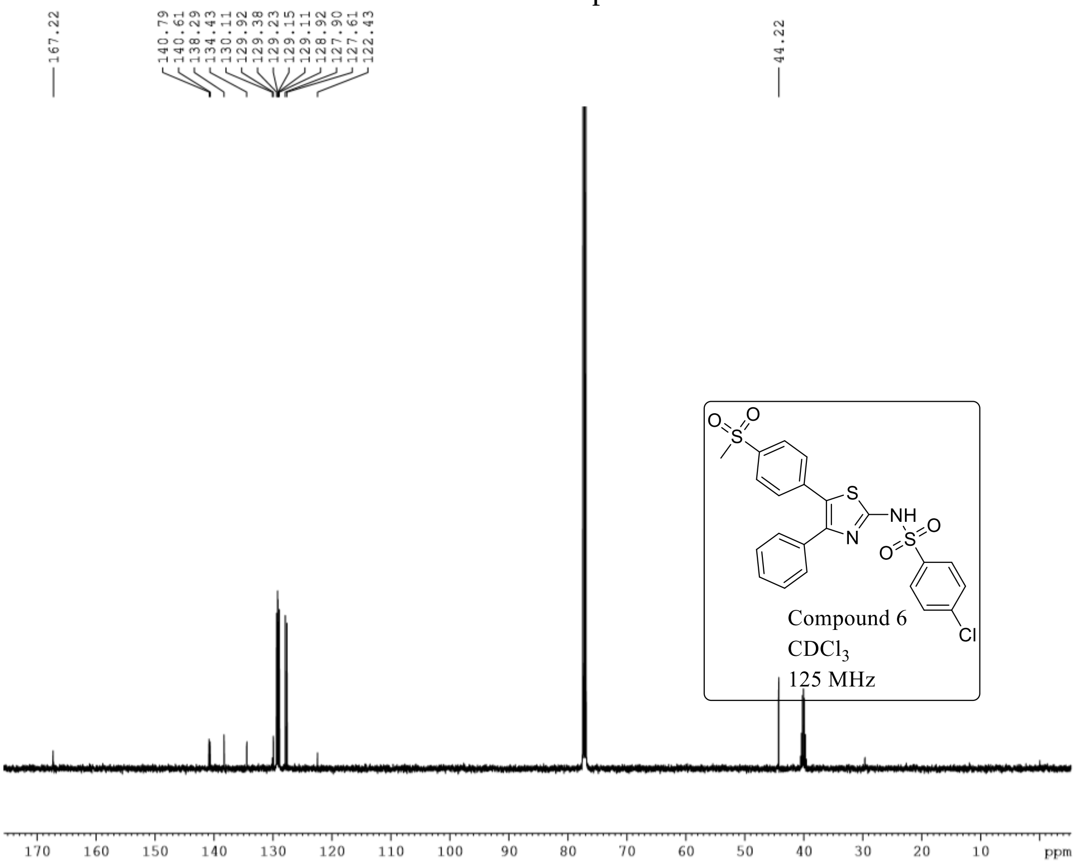

^{13}C NMR of compound 6

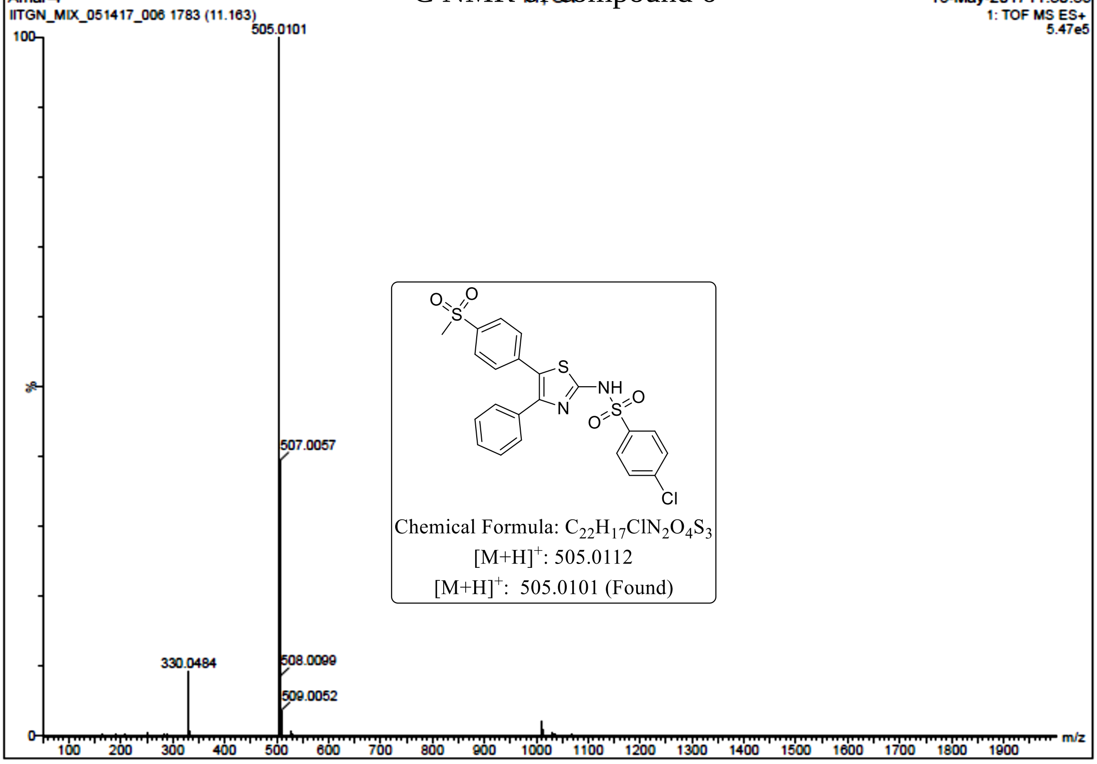

LC-ESIMS of Compound 6

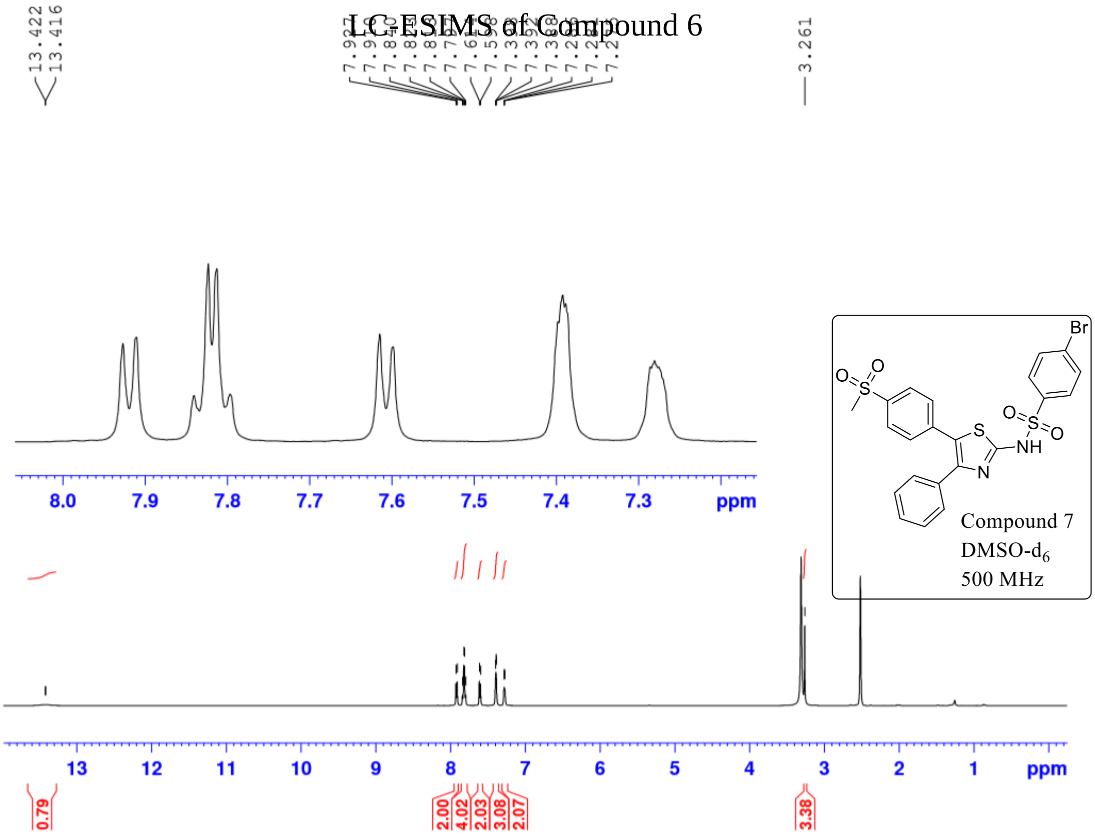

^{1}H NMR of compound

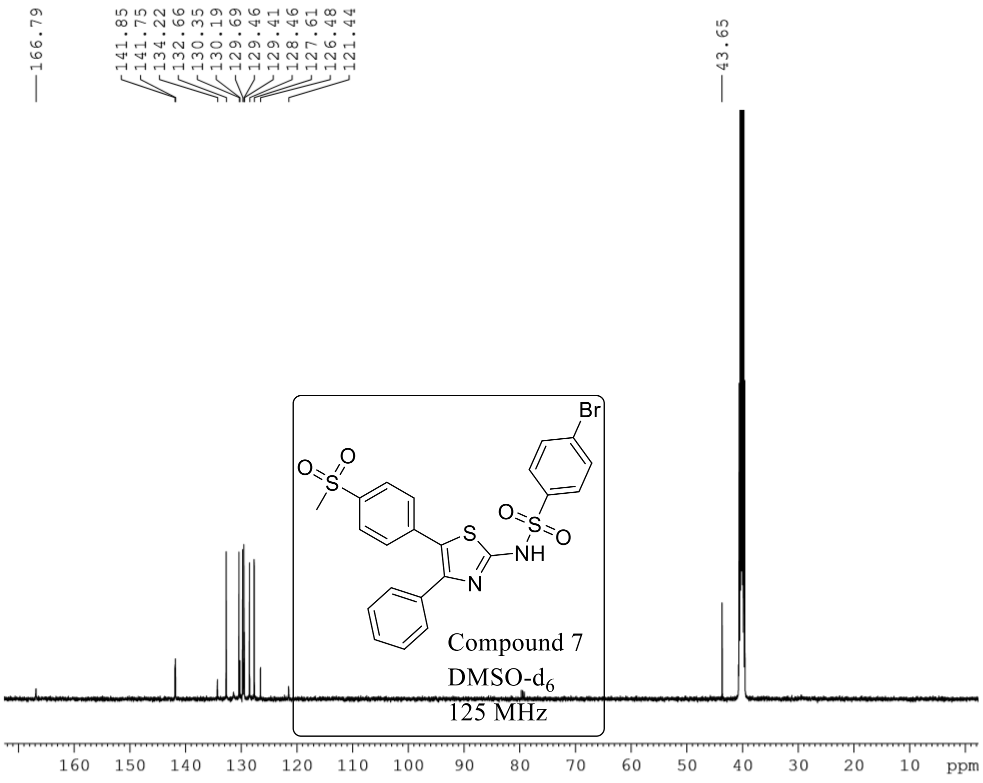

^{13}C NMR of compound 7

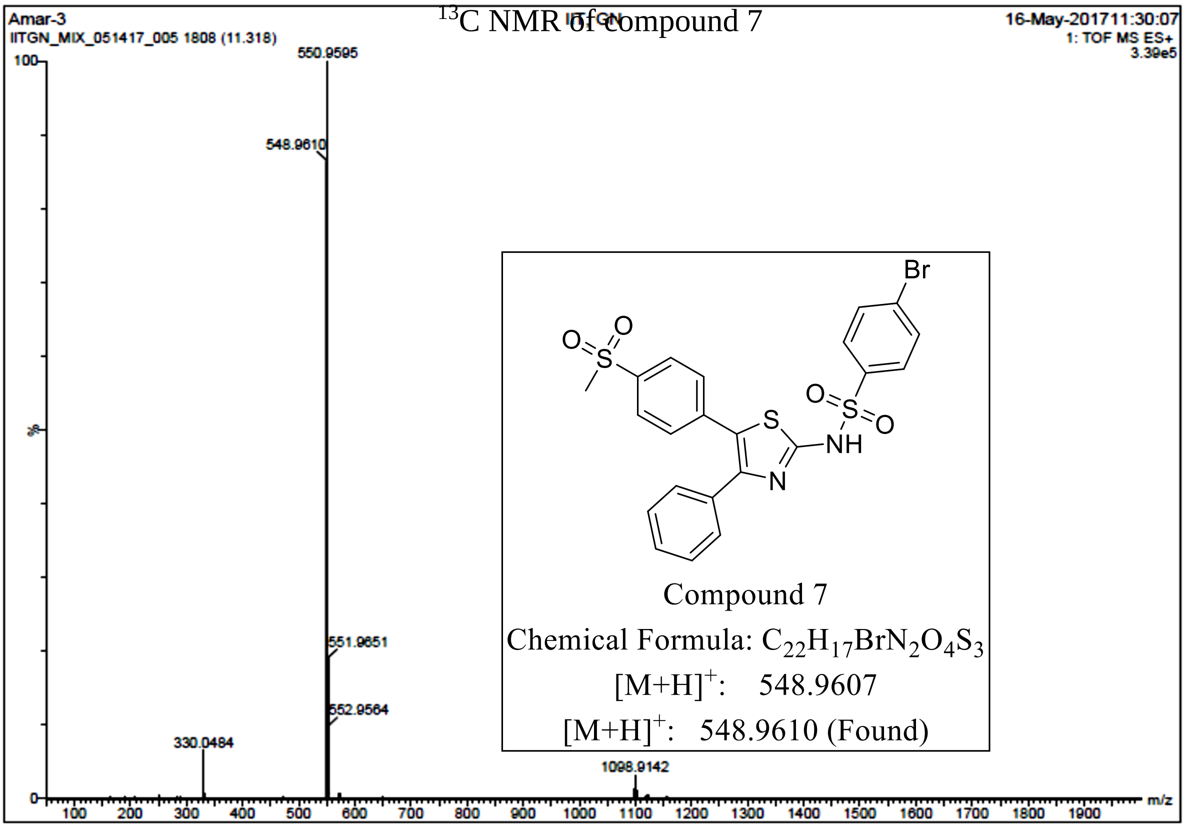

LC-ESIMS of Compound 7

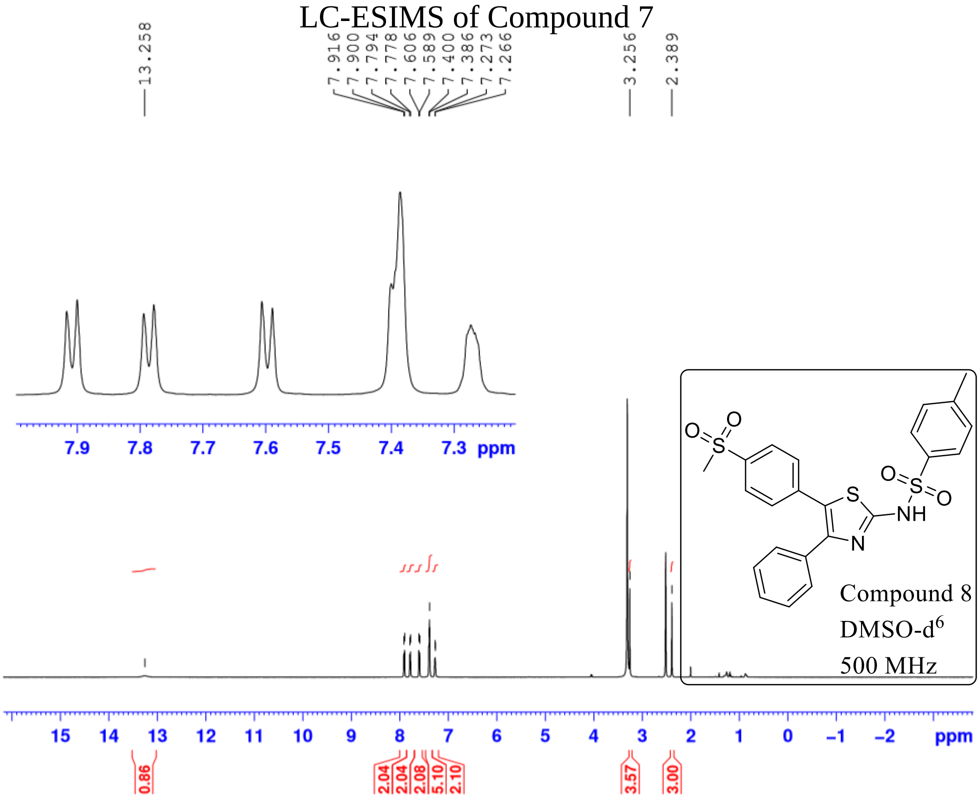

^{1}H NMR of compound 8

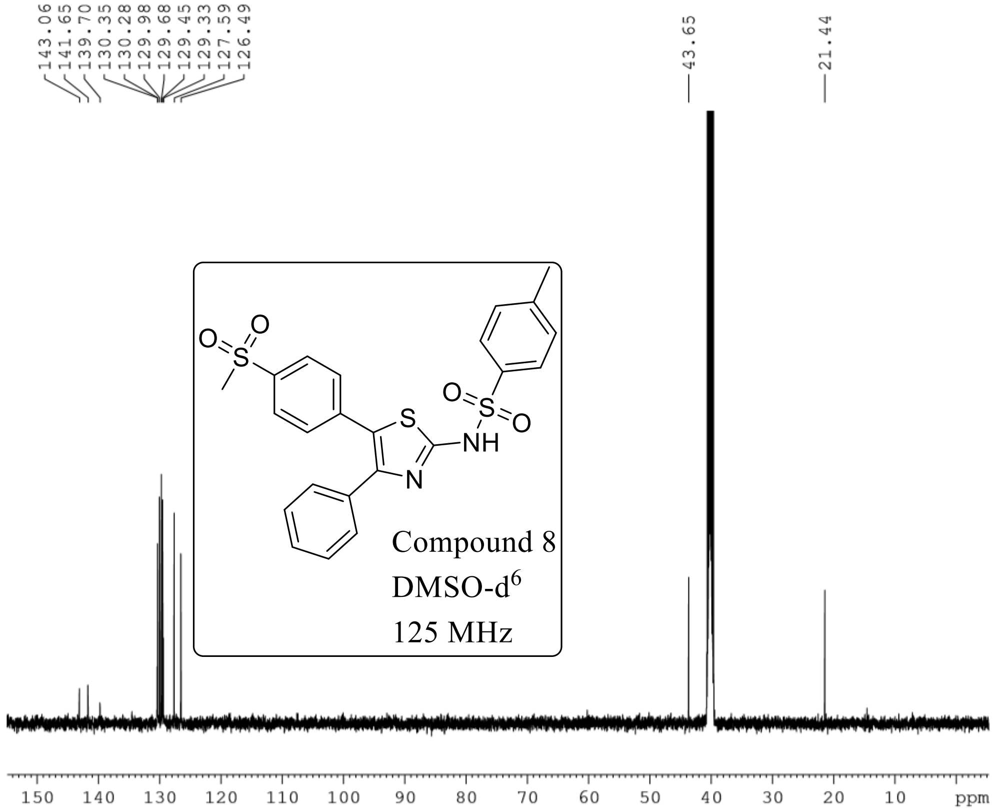

^{13}C NMR of compound 8

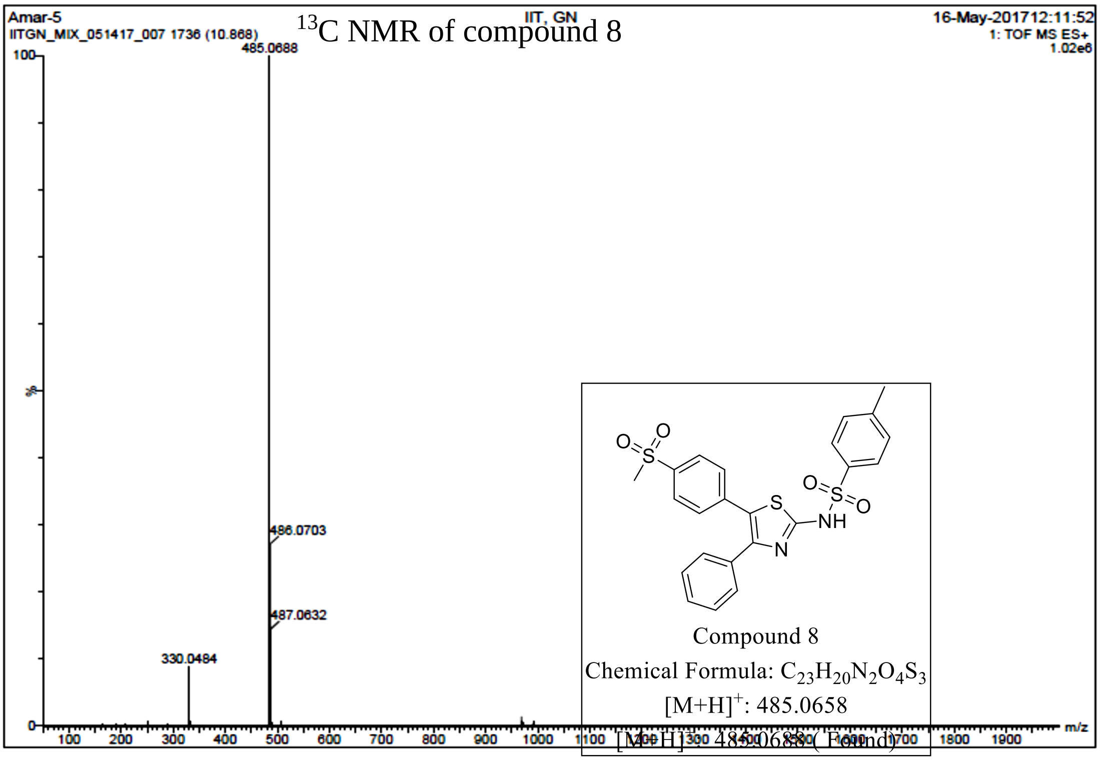

LC-ESIMS of Compound 8

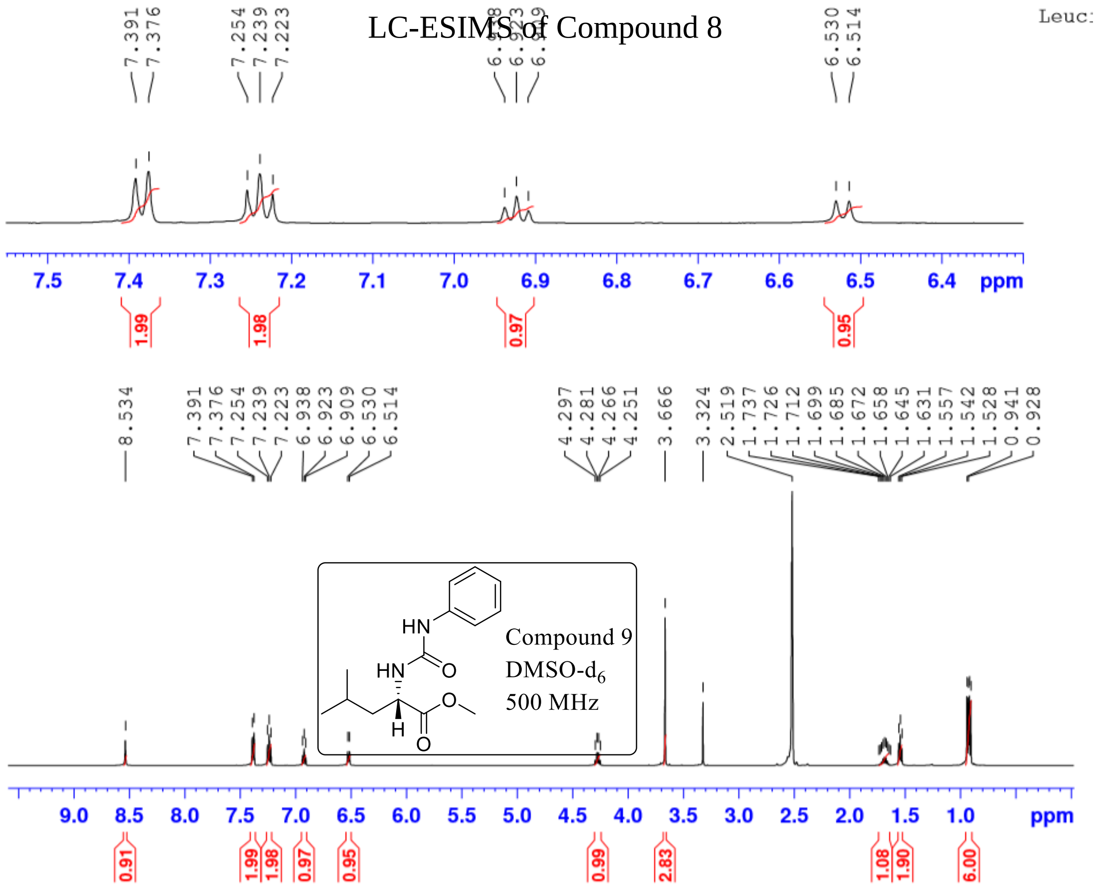

^{1}H NMR of compound 9

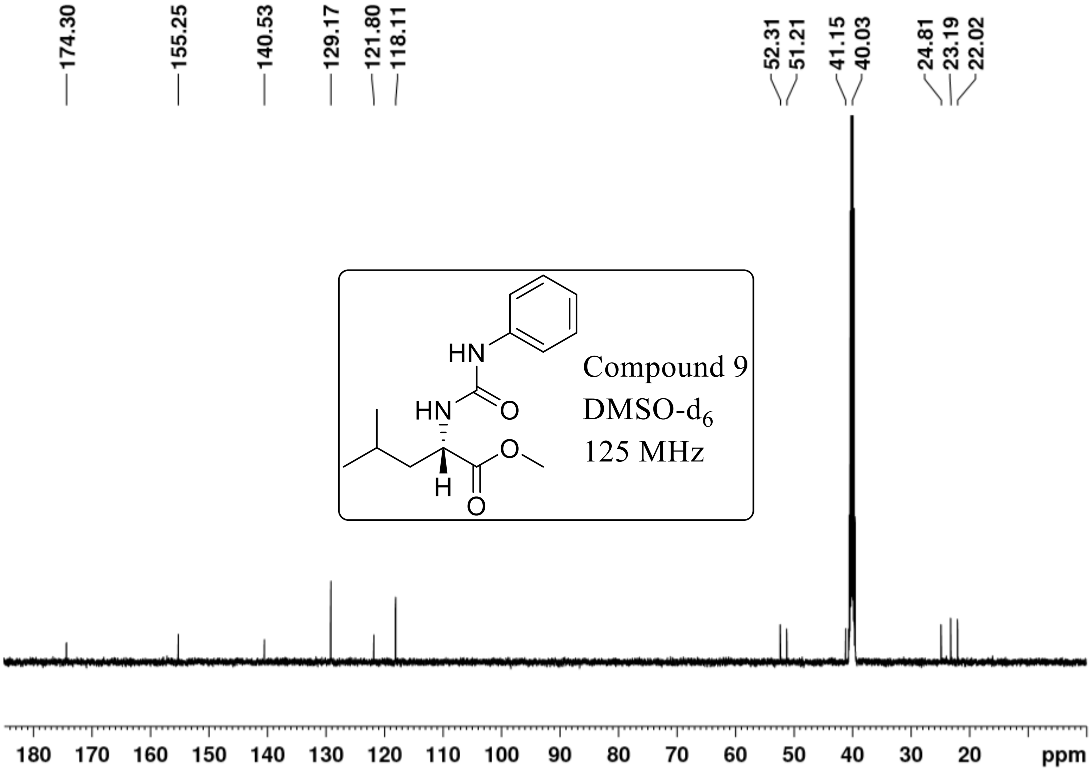

^{13}C NMR of compound 9

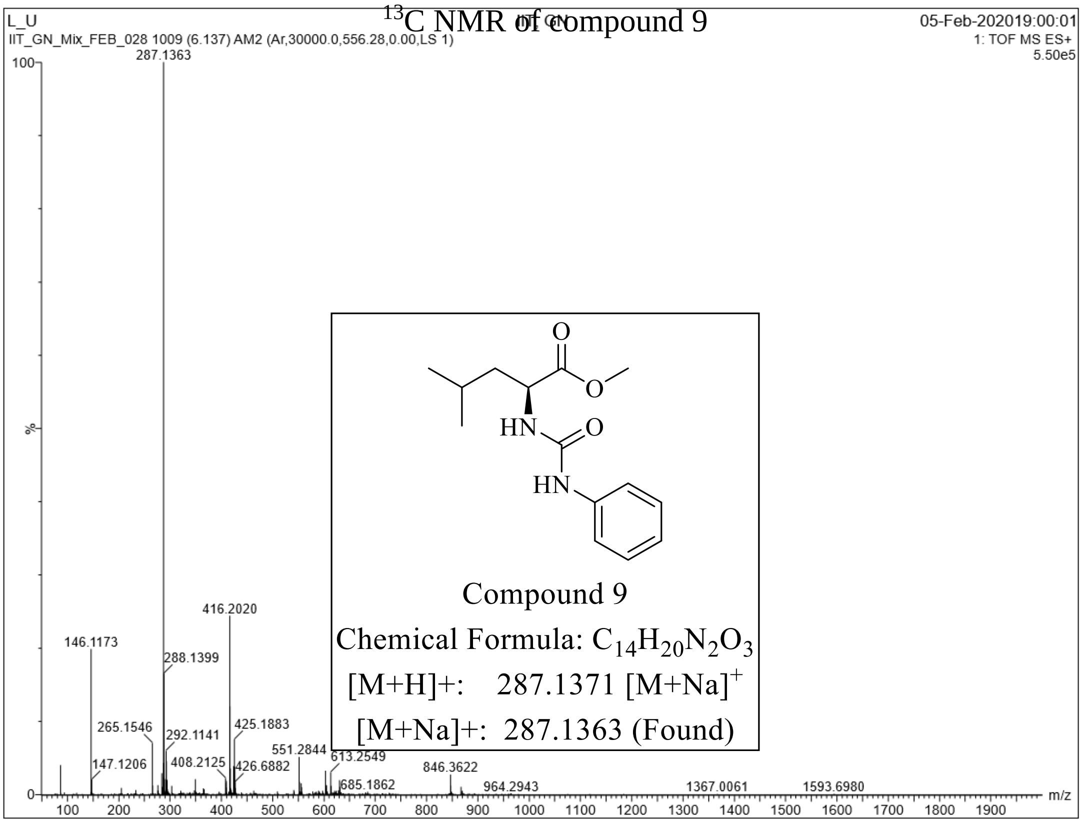

LC-ESIMS of Compound 9

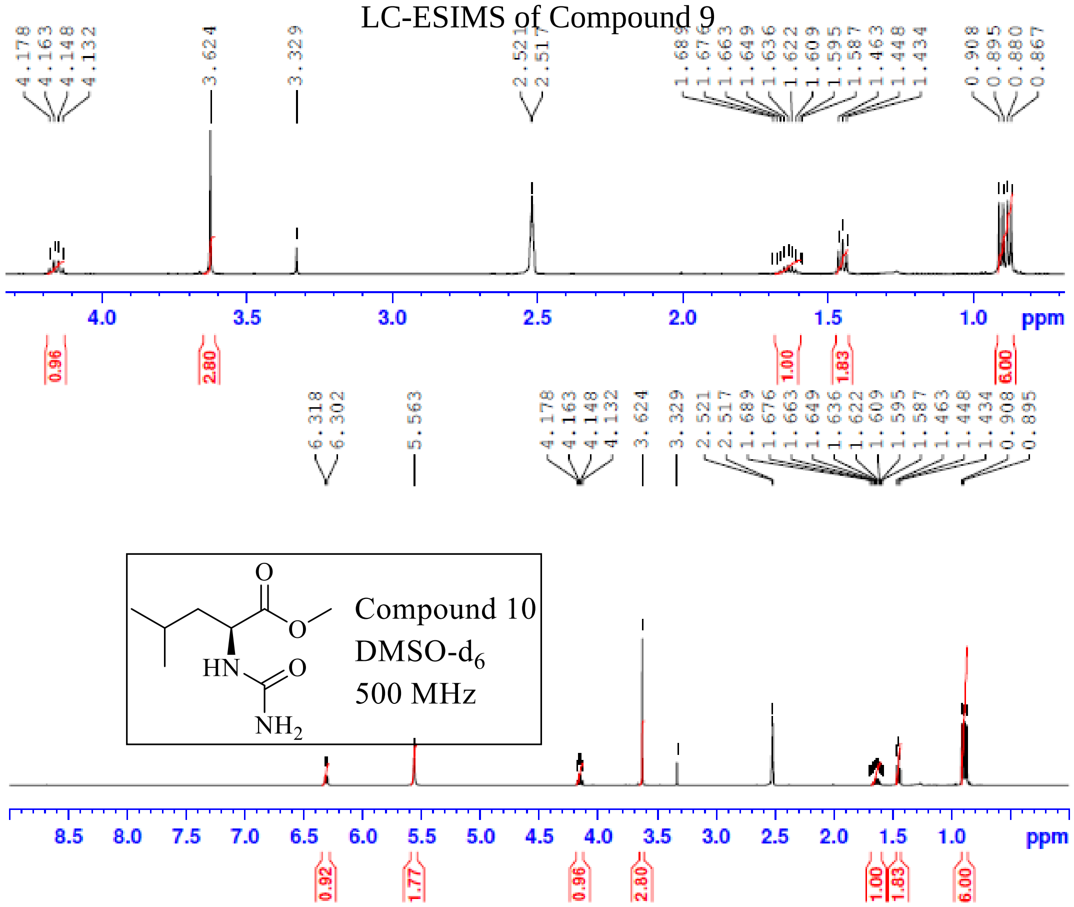

^{1}H NMR of compound 10

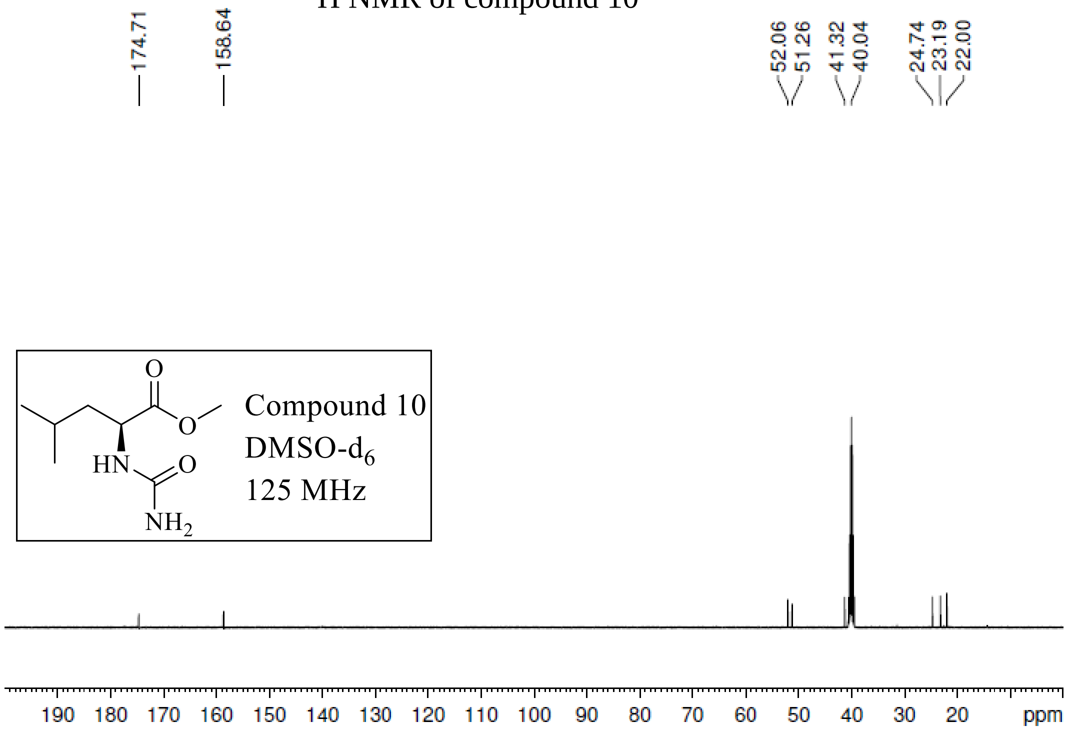

^{13}C NMR of compound 10

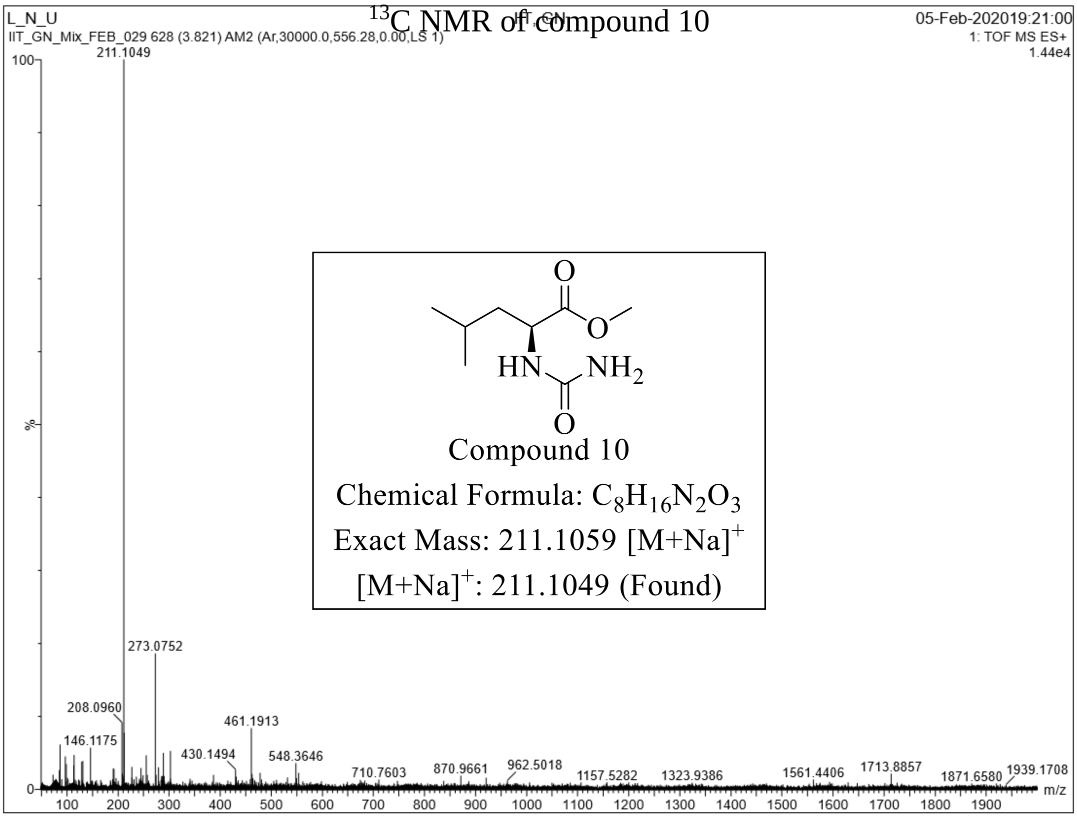

LC-ESIMS of Compound 10

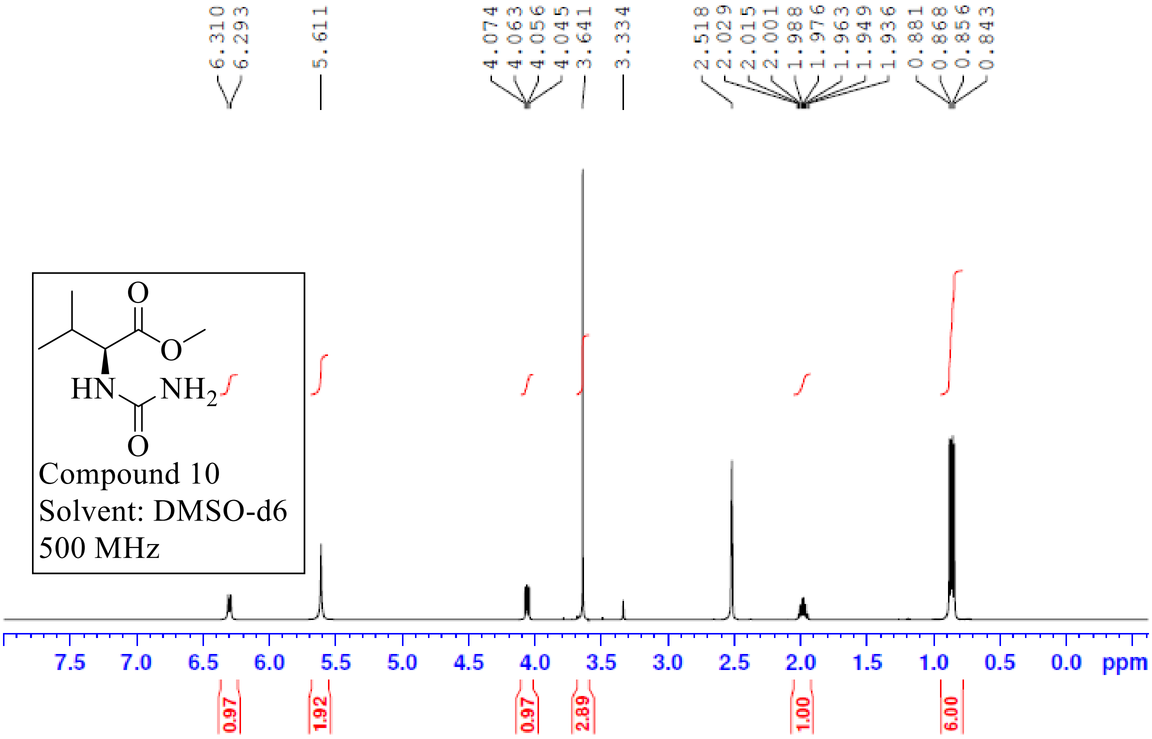

^{1}H NMR of compound 11

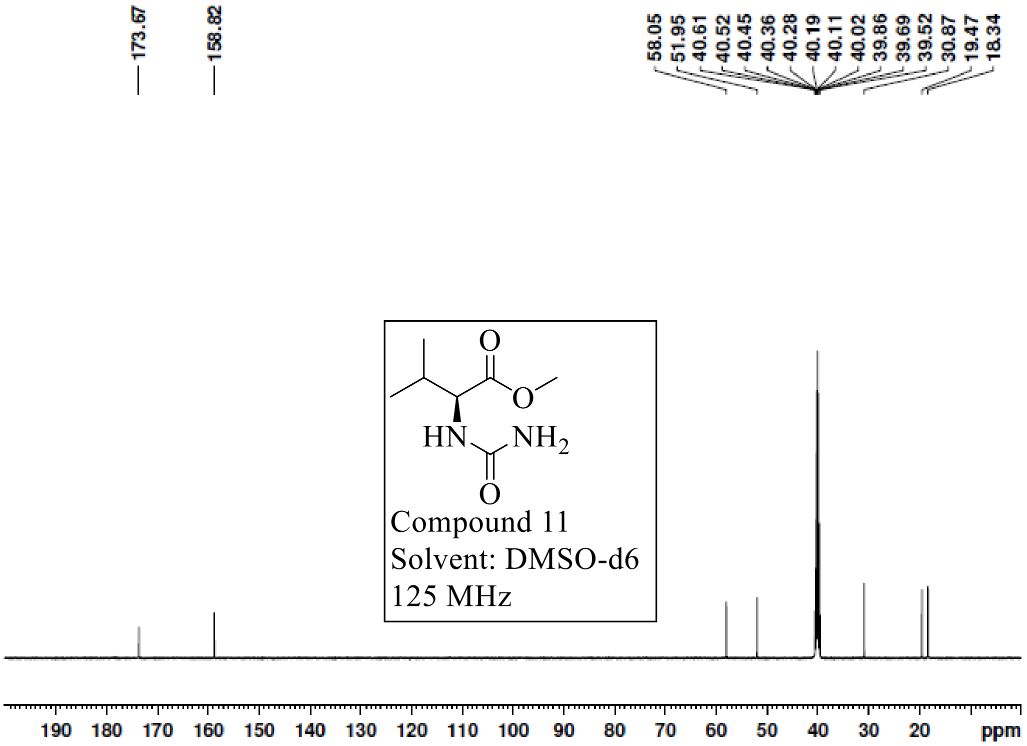

^{13}C of compound 11

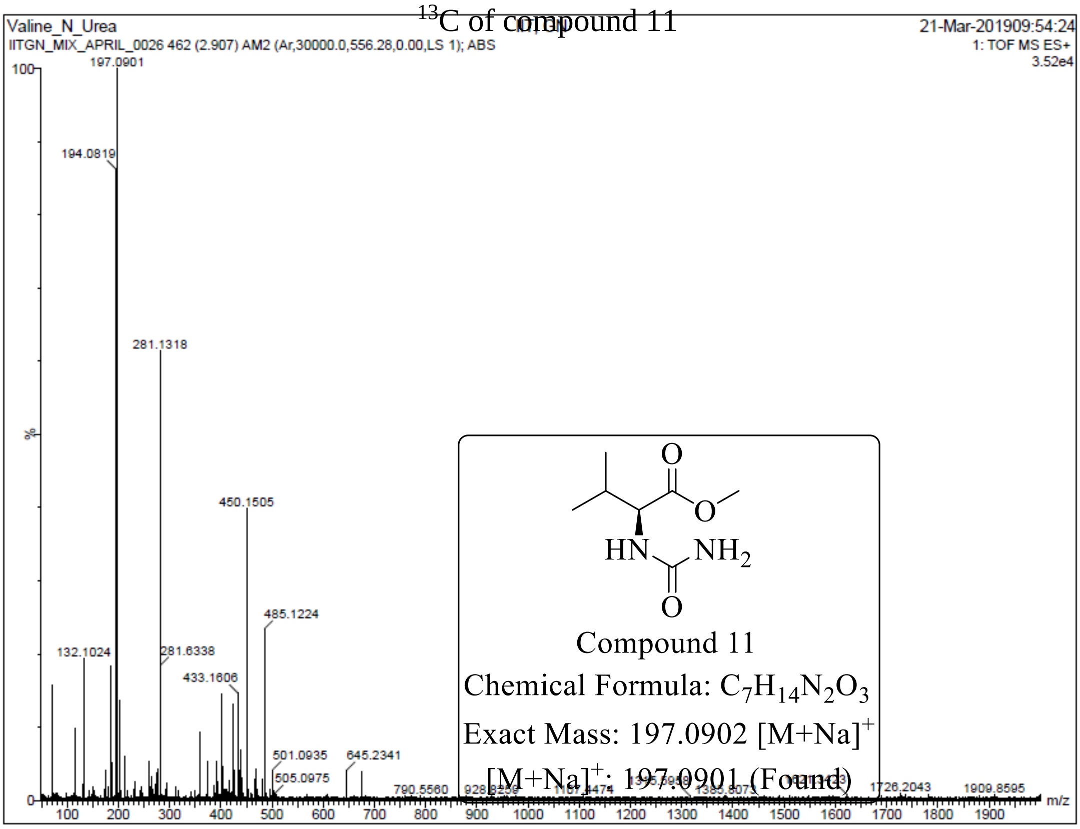

LC-ESIMS of Compound 11

Figure S1. Characterization of all synthesized compounds (1 to 11) by different spectroscopic techniques like ^{1}H NMR, ^{13}C NMR, and LC-MS mass spectrometry.

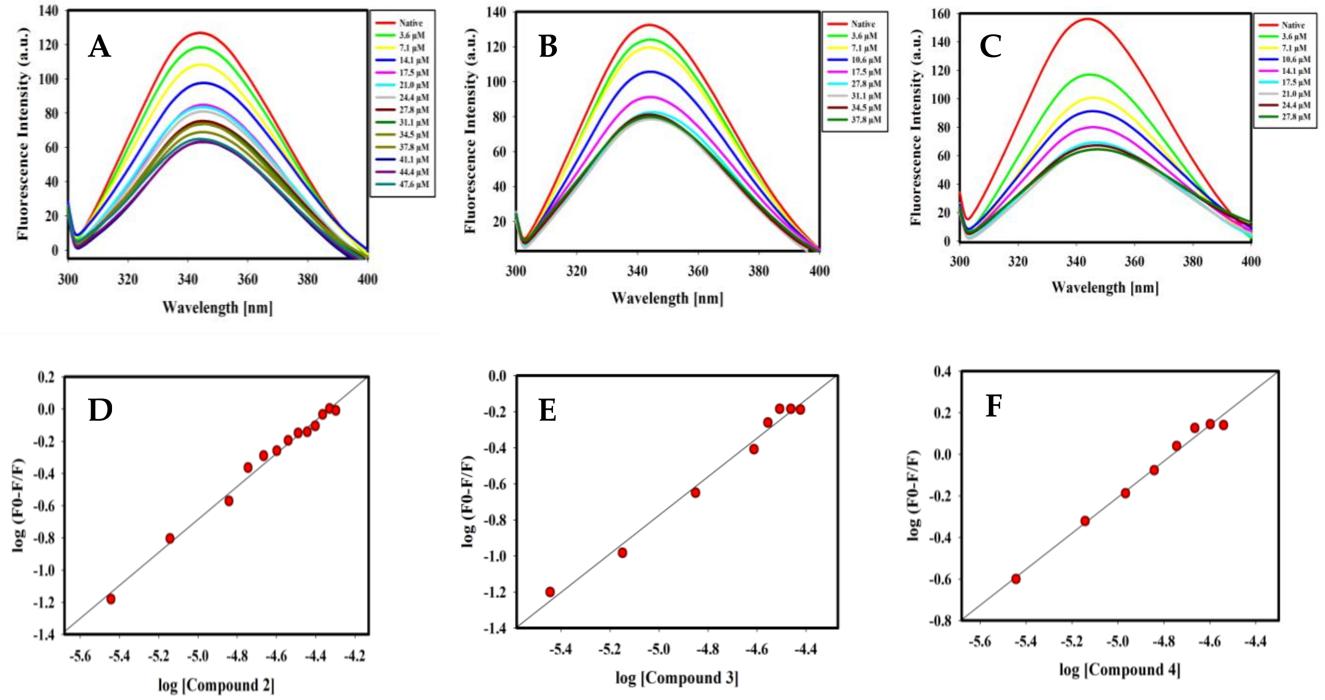

Figure S2. Binding studies of compound 2,3 and 4 with SphK1. Fluorescence emission spectra representing SphK1 quenching on the addition of an increasing amount of (**A**) compound 2 (0–47.6 µM), (**B**) compound 3 (0–37.8 µM) and (**C**) compound 4 (0–27.8 µM). SphK1 was excited at 280 nm and emission spectra were recorded in the range of 300–400 nm. Modified Stern-Volmer plot showing quenching of SphK1 fluorescence with increasing concentration of (**D**) compound 2, (**E**) compound 3 and (**F**) compound 4. The SV plot was used to calculate binding constant (*K*a) and the number of binding sites (*n*).

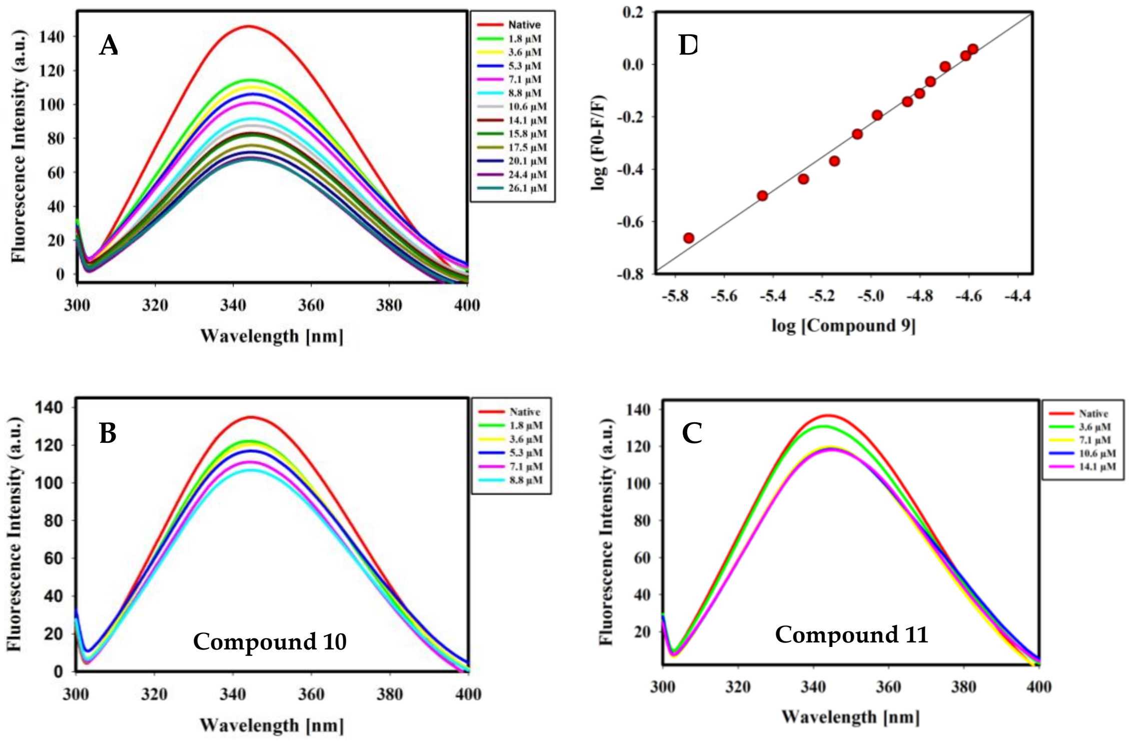

Figure S3. Binding studies of compound 9,10 and 11 with SphK1. Fluorescence emission spectra representing SphK1 quenching on the addition of an increasing amount of (**A**) compound 9 (0–26.1 μM), (**B**) compound 10 (0–8.8 μM) and(**C**) compound 11 (0–14.1 μM). SphK1 was excited at 280 nm and emission spectra were recorded in the range of 300–400 nm. (**D**) Modified Stern-Volmer plot showing quenching of SphK1 fluorescence with increasing concentration of compound 9. The SV plot was used to calculate binding constant (*K*a) and the number of binding sites (*n*).

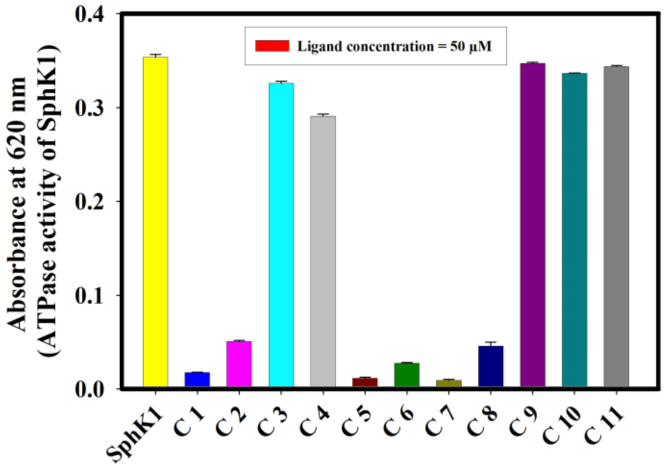

Figure S4. Screening of compounds for evaluation of their inhibitory potential against SphK1 by malachite green-based ATPase inhibition assay. The inorganic phosphate released by ATPase activity of SphK1 forms a green complex with the malachite green that absorbs at 620 nm.

Table S1. List of different non-covalent interactions between compounds and SphK1 interacting residues.

S. No.	Interaction type	Participating residues of SphK1 with compounds					
		**Compound 1**	**Compound 5**	**Compound 6**	**Compound 7**	**Compound 8**	***PF-543**
**1.**	**Hydrogen bonds**	Asp 178	-	Thr 196	Thr 196	Thr 196	Asp 178, Thr 196, His 311
**2.**	**van der Waals**	Met 272, Leu 167, Leu 268, Asp 81, Val 177, Ile 174, Thr 196, Phe 173, Leu 299	Thr 196, Phe 303, Phe 173, Leu 259, Leu 319, His 311	Phe 303, Leu 259, Leu 200, Leu 302, Leu 319, Phe 288, His 311, Ala 274, Leu	Met 272, Val 177, Phe 288, His 311, Leu 319, Leu 259, Leu 302, Val 290	Phe 303, Leu 200, Leu 259, Leu 302, Leu 319,Phe 288, His 311, Ala 274, Leu	Phe 288, Leu 259, Leu 302, Leu 261, Val 290, Met 306, Ala 170, Ala 339, Gly 342,

				261, Met 272, Val 177		261, Met 272, Val 177	Leu 167, Ser 168
**3.**	**Pi-Sigma**	-	-	-	-	-	Phe 192
**4.**	**Pi-Alkyl**	Ala 115, Leu 319, His 311, Phe 288, Met 306, Leu 259	Ile 174, Val 177, Met 272, Leu 268, Leu 302, Met 306	Ala 115, Leu 167, Leu 268, Ile 174, Leu 299	Leu 268, Ile 174, Met 306, Leu 200, Leu 299	Ala 115, Leu 167, Leu 268, Ile 174, Leu 299	Ala 274, Leu 319, Leu 200, Leu 299, Phe 173, Val 177, Ile 174, Leu 268
**5.**	**Pi-Sulfur**	Phe 303		Met 306	Phe 303	Met 306	Met 272
**6.**	**Pi-Anion**	-	Asp 178	Asp 178	Asp 178	Asp 178	-
**7.**	**Pi- Pi Stacked**	Phe 192	Phe 192	Phe 173, Phe 192	Phe 173, Phe 192	Phe 173, Phe 192	Phe 303

*PF-543 is used as a reference compound in docking experiments for comparision of the binding pattern observed for compounds under investigation.
